# Food and medicine homology: a potential nutritional intervention strategy for post-acute COVID-19 syndrome

**DOI:** 10.3389/fphar.2025.1588037

**Published:** 2025-07-03

**Authors:** Zhan Li, Yingqi Liu, Wei Ding, Yue Liu, Wang Li, Shanshan Guan, Xianjun Liu, Guizhen Wang, Qiong Liu, Chunwa Jiang, Xinli Peng, Hao Li, Zhandong Li, Jing Li

**Affiliations:** ^1^ School of Biological and Food Engineering, Jilin Engineering Normal University, Changchun, China; ^2^ School of Chemistry and Life Sciences, Changchun University of Technology, Changchun, China; ^3^ Engineering Research Center of Edible and Medicinal Fungi, Ministry of Education, Jilin Agricultural University, Changchun, China; ^4^ School of Computer Science, Baicheng Normal University, Baicheng, China

**Keywords:** food and medicine homology, post-acute COVID-19 syndrome, food intervention, food safety, food development

## Abstract

After recovering from severe acute respiratory syndrome of coronavirus 2 (SARS-CoV-2) acute infection, some patients with corona virus disease 2019 (COVID-19) still are affected by post-acute COVID-19 syndrome (PACS). Traditional Chinese medicine (TCM) has played an important role in the recovery period of COVID-19. As a unique type of Chinese botanical drugs, foods with property of food and medicine homology (FMH) has the dual characteristics of drug and food, which has advantages over usual drugs in safety and daily application. This review analyzed a total of 290 peer-reviewed publications on the progress of dozens of formulas and single botanical drugs, which were systematically collected from the electronic scientific databases, including PubMed, Web of Science, and China National Knowledge Infrastructure (CNKI), as well as other literature sources, such as classic Chinese medicine books. The symptoms of PACS and the advancements in the application of FMH foods in PACS intervention are summarized, and the challenges in the regulatory characteristics and food safety are further discussed. It is expected that the application of FMH foods would bring new opportunity for the treatment and daily intervention of PACS, and this review provides a foundation for the development of PACS intervention foods.

## Graphical Abstract

**Figure:**
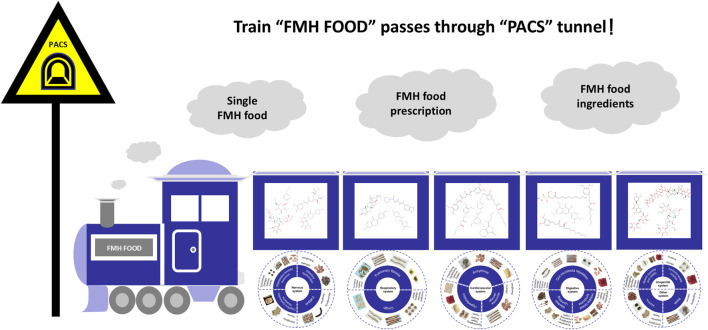

## 1 Introduction

In the past years, the corona virus disease 2019 (COVID-19) pandemic has caused severe infection worldwide. On 5 May 2023, the World Health Organization (WHO) announced that COVID-19 was no longer a Public Health Emergency of International Concern (PHEIC) and transitioned to long-term management (Harris, 2023; Parums, 2023). The sequelae of COVID-19 are an important factor threatening human health in the post-epidemic era. Many studies have shown that although some COVID-19 patients have recovered from acute viral infection, multiple organs and systems are still be affected in the long term, and these symptoms are designated as post-acute COVID-19 syndrome (PACS) (Davis et al., 2023; Szabo et al., 2023), which is widely defined as “new signs and symptoms occurring 4–8 weeks after recovering from acute stage of COVID-19” (Szabo et al., 2023). Statistically, about 65 million people around the world are affected by PACS, with a prevalence of about 9%–63%, which is six times higher than those of similar post-viral infections (Davis et al., 2023; Lippi et al., 2023). PACS is susceptible to all age groups, and is more common in mild acute patients (The Lancet, 2023). PACS is characterized as having systemic and long-term symptoms involved in the nervous system, respiratory system, cardiovascular system, immune system, digestive system, and urogenital system (Gang et al., 2022; Brevini et al., 2023). At present, more than 200 symptoms have been found to be related to PACS, such as fatigue, insomnia, anxiety, sexual dysfunction, liver and kidney damage, myocardial injury, arrhythmia, hypertension, etc. (Lippi et al., 2023; The Lancet, 2023; Zaidi and Dehgani-Mobaraki, 2024). These medical disorders are probably caused by the extensive expression of angiotensin-converting enzyme 2 (ACE2) receptor (i.e., facilitator of SARS-CoV-2 entering cells) and many dysfunctional biological processes (such as chronic inflammation, endocrine disorders, immune disorders, tissue damage, tissue hypoxia, dysbacteriosis, coagulation and endothelial abnormalities, and neurological signal dysfunction) (Brevini et al., 2023; Davis et al., 2023; Szabo et al., 2023; Turner et al., 2023; Zaidi and Dehgani-Mobaraki, 2024).

The foods with property of food and medicine homology (FMH) refer to traditional Chinese medicines (TCMs) with both food and medicine functions. The FMH foods take into account the dual characteristics of both medicine and food, and are in line with the food requirements of modern healthy life (Hou and Jiang, 2013; Ma et al., 2022). Daily consumption of FMH foods can enhance body function and prevent diseases, and is considered a mild potential intervention different from drug therapy (Hu et al., 2022; Ng et al., 2023). The rich active ingredients in the FMH foods, such as flavonoids, saponins, alkaloids, and organic acids, are the foundation of significant PACS intervention activity (Hu et al., 2022; Lu et al., 2022), and the characteristics of “multi-target, multi-metabolite, and multi-pathway” of FMH foods provide a systematic efficacy in the treatment of many medical disorders (Wang et al., 2012). For example, the resveratrol in Sangshen (*Morus alba* L.) is considered effective for pulmonary fibrosis (PF), fatigue, and arrhythmia (Wang J. et al., 2022; Ma et al., 2023; Zhang X. et al., 2023), and the 6-gingerol, 8-gingerol, 10-gingerol, and 6-shogaol in Shengjiang (*Zingiber officinale* Rosc.) can cope with nausea and vomiting (Pertz et al., 2011; Haniadka et al., 2012).

There are strong linkages among the multi-system symptoms, complex pathogenesis of PACS, and the “multi-metabolites, multi-target, and multi-channel” treatment mechanism of FMH foods (Su et al., 2023). To date, numerous investigations have demonstrated the efficiency of FMH foods on the intervention of PACS-related symptoms. However, a systemic review of the mechanisms regulating the therapeutic effects of FMH foods is still lacking. The goals of our review were to: (1) comprehensively summarize the symptoms of PACS and the advancements in the application of FMH foods in PACS intervention, and (2) discuss the challenges in the regulatory characteristics and FHM food safety. We selected the total of 105 FMH foods published by the National Health Commission of China and the FMH prescriptions in ancient books of TCM as the drug objects, and identified the characteristic symptoms of different systems of PACS as the disease objects. These drug and disease objects were used as key words to conduct literature search for relevant publications in the PubMed, Web of Science, and China National Knowledge Infrastructure (CNKI) databases, as well as other literature sources. It is expected that the application of FMH foods would bring new opportunities for the treatment and daily intervention of PACS, and this review provides a foundation for the development of PACS intervention foods.

## 2 FMH foods and their clinical applications

The modern definition of FMH foods refers to TCMs that are consumed long-term as part of the diet (in limited doses and specific combinations), and are officially recognized by the national authorities as both edible and medicinal, with a long history of dietary use (Mou et al., 2023). The FMH foods can be divided into two categories according to the number of food in the dietary material, “single FMH food” contains only one food, and “FMH food prescription” refers to the edible products with different forms of dietary components (Zhai, 2018). The ideological origin of FMH can be traced back to the allusion of “*Shen Nong Tastes Herbs,*” which was widely recognized in the Xia, Shang, and Zhou Dynasties with the applications of fire and the progress of cooking technology (Ji et al., 2023). From ancient times to the present, the idea of FMH is commonly practiced in diet therapy and the application of medicinal diet (Fan et al., 2016). Medicinal diet refers to the rational combination of different drugs and foods under the guidance of the theory of TCM, and it is made by both traditional and modern scientific processing technologies, with unique color, fragrance, taste, shape, and effect (Tan, 2017). The concept of medicinal diet started in the Qin and Han Dynasties, further developed in the Jin and Tang Dynasties, flourished in the Song and Yuan Dynasties, and well established in the Ming and Qing Dynasties (Tan, 2017). Medicinal diet prescriptions include “pure food prescription” (i.e., “single FMH food” is considered food) and “medicine and food combined prescription.” Here, the “pure food prescription” is classified as “FMH food prescription” (Zhai, 2018).

It is worth noting that in the development of FMH food prescription, it is necessary to replace no-FMH food in the “medicine and food combined prescription” with FMH food (Duan et al., 2018). Many FMH food prescriptions have been recorded in the descriptions of TCM written by scholars in the past dynasties, showing curative effects in the treatment of PACS related symptoms, such as insomnia, depression and sore throat. For example, Gancao Decoction (Gancao; *Glycyrrhiza uralensis* Fisch.) and Jiegeng Decoction (Gancao and Jiegeng; *Platycodon grandiflorum* (Jacq.) A. DC.) are used to treat pharyngalgia (Zhou and Hu, 2017). Fuling Xingren Gancao Decoction (Fuling; *Poria cocos* (Schw.) Wolf, Xingren; *Prunus armeniaca* L. var. *ansu* Maxim, and Gancao) are used to treat chest distress and cough (Yao et al., 2021). Furthermore, modified versions of Fuling Xingren Gancao Decoction have been widely used in several well-known hospitals in China to treat symptoms, such as cough, chest tightness, and shortness of breath, with sound therapeutic effects (Yao et al., 2021; Yu et al., 2024; Wang L. et al., 2022; Li and Lin, 2020). Ganmai Dazao Decoction (Gancao and Dazao; *Ziziphus jujuba* Mill.) is used to treat insomnia and neurosis (Zhou and Hu, 2017), while Ye Tianshi, an ancient renowned TCM physician, used secondary formulas of Ganmai Dazao Decoction to treat mental illnesses (Guo et al., 2023). The results of modern clinical research reveal significant effect of Ganmai Dazao Decoction, combined with Guishen Pill (Shanyao; *Dioscorea opposita* Thunb., Fuling, Danggui; *Angelica sinensis* (Oliv.) Diels., and Gouqizi; *Lycium barbarum* L., etc.) on the treatment of perimenopausal sleep disorders, which is likely attributed to the regulation of sex hormones, neurotransmitters, and oxidative stress (Pan et al., 2023). Moreover, the combination of Ganmai Dazao Decoction and Suanzaoren Decoction (Suanzaoren; *Z. jujuba* Mill. var*. spinosa* (Bunge) Hu ex H. F. Chou) has also been demonstrated to be effective for the treatment of insomnia (Li, 2019). A list of representative FMH food prescriptions related PACS are provided in Table 1.

**TABLE 1:** Examples of FMH food prescription intervention for PACS^a^.

Name	FMH food	Latin Name/Common Name	Source	Dynasty	Author	Symptom/Function	Reference
Ganmai Dazao Decoction	Gancao Dazao	*Glycyrrhiza glabra* L. *Ziziphus jujuba* Mill.	Synopsis of the Golden Chamber	Han	Zhang Zhongjing	Tranquilization, Insomnia, neurosis	Zhou and Hu (2017)
Baihe Jizihang Decoction	Baihe Danhuang	*Lilium lancifolium* Thunb. Yolk	Synopsis of the Golden Chamber	Han	Zhang Zhongjing	Insomnia	Zhou and Hu (2017)
Fuling Xingren Gancao Decoction	Fuling Xingren, Gancao	*Poria cocos* (Schw.) Wolf *Prunus armeniaca* L. var. *ansu* Maxim *Glycyrrhiza glabra* L.	Synopsis of the Golden Chamber	Han	Zhang Zhongjing	Chest distress, Cough	Yao et al. (2021)
Jiegeng Decoction	Gancao, Jiegeng	*Glycyrrhiza glabra* L. *Platycodon grandiflorum* (Jacq.) A. DC.	Treatise on Febrile Diseases	Han	Zhang Zhongjing	Pharyngalgia	Zhou and Hu (2017)
Gancao Ganjiang Decoction	Gancao Ganjiang	*Glycyrrhiza glabra* L. *Zingiber officinale* Rosc.	Treatise on Febrile Diseases	Han	Zhang Zhongjing	Respiratory diseases	Niu et al. (2021)
Zhufu Decoction	Zhupi Damifen Fengmi	Pigskin Rice powder *Apis cerana* Fabricius	Treatise on Febrile Diseases	Han	Zhang Zhongjing	Pharyngalgia	Zhou and Hu (2017)
–	Huajiao Xingren Dazao	*Zanthoxylum bungeanum* Maxim. *Prunus armeniaca* L. var. *ansu* Maxim. *Ziziphus jujuba* Mill.	Prescriptions for Emergent Reference	Jin	Ge Hong	Cough	Fan et al. (2016)
–	Chixiaodou Guadi	*Vigna umbellata* Ohwi et Ohashi Calyx Cucumis	Prescriptions for Emergent Reference	Jin	Ge Hong	Headache, Loss of appetite	Fan et al. (2016)
–	Biba	*Piper longum* L.	Prescriptions for Emergent Reference	Jin	Ge Hong	Nausea	Fan et al. (2016)
–	Hujiao Ganjiang Jupi Jiyu	*Piper nigrum* L. *Zingiber officinale* Rosc. *Citrus reticulata* Blanco *Carassius auratus*	Prescriptions for Emergent Reference	Jin	Ge Hong	Weakness of the spleen and the stomach	Fan et al. (2016)
–	Muli Gancao Rougui	*Ostrea gigas* Thunberg *Glycyrrhiza glabra* L. *Cinnamomum cassia* Presl	Prescriptions for Emergent Reference	Jin	Ge Hong	Stomachache	Fan et al. (2016)
	Shengjiang Ganzhe	*Zingiber officinale* Rosc. *Saccharum officinarum* L.	Prescriptions for Emergent Reference	Jin	Ge Hong	Vomit	Fan et al. (2016)
Jiang Miyin	Shengjiang	*Zingiber officinale* Rosc.	Sheng Ji Zong Lu	Song	Zhao Ji	Cough, Vomit	Ji and Yang (2023)
Hujiao Miyin	Hujiao	*Piper nigrum* L.	Sheng Ji Zong Lu	Song	Zhao Ji	Eliminating phlegm	Ji and Yang (2023)
Zisu Miyin	Zisu	*Perilla frutescens* (L.) Britt.	Sheng Ji Zong Lu	Song	Zhao Ji	Removing food retention	Ji and Yang (2023)
Zisu Mugua Miyin	Zisu Mugua	*Perilla frutescens* (L.) Britt. *Chaenomeles speciosa* (Sweet) Nakai	Sheng Ji Zong Lu	Song	Zhao Ji	Diarrhea	Ji and Yang (2023)
Suanzaoren Zou	Suanzaoren	*Ziziphus jujuba* Mill. var*. spinosa* (Bunge) Hu ex H. F. Chou	Essentials of Diet	Yuan	Hu Sihui	Insomnia	Zhang et al. (2019)
Shanyao Decoction	Shanyao Xingren	*Dioscorea opposita* Thunb. *Prunus armeniaca* L. var. *ansu* Maxim	Essentials of Diet	Yuan	Hu Sihui	Moistening lung	Wang et al. (2020c)
Xingshuang Decoction	Xingren	*Prunus armeniaca* L. var. *ansu* Maxim	Essentials of Diet	Yuan	Hu Sihui	Cough	Wang et al. (2020a)
Baihe Zou	Baihe	*Lilium lancifolium* Thunb.	Compendium of Materia Medica	Ming	Li Shizhen	Insomnia, Tranquilization	Zhang et al. (2019)
Yiyiren Zou	Yiyiren	*Coix lacryma-jobi* L. var. *mayuen.* (Roman.) Stapf	Compendium of Materia Medica	Ming	Li Shizhen	Reconciling gastrointestinal	Zhou et al. (2020)
Sangshen Gao	Sangshen	*Morus alba* L.	Sui Xi Ju Yin Shi Pu	Qing	Wang Shixiong	Insomnia	Zhang et al. (2019)

^a^ Data are retrieved from Traditional Chinese Medicine Systems Pharmacology Database and Analysis Platform (TCMSP) (https://www.91tcmsp.com/#/home).

The history of FMH food can be traced back to the “*The Classic of Poetry,*” the oldest existing collection of Chinese poetry, as evidenced by the desciptions of Gouqizi and Huajiao (*Zanthoxylum bungeanum* Maxim.) (Mou et al., 2023). There are also records of “five grains, five livestock, five fruits, and five vegetables” in “*Inner Canon of Huangdi,*” one of the most important foundation texts of TCM. It is worth explaining that in modern times, “five grains” means grains, “five fruits” represents fruits, “five livestock” indicats meat, and “five vegetables” stands for vegetables. These concepts represent the most precise definition of FMH food and are also the earliest records in the literature (Mou et al., 2023). FMH food is the concentrated expression of modern FMH idea (Xie et al., 2020). From 2002 to 2024, National Health and Health Commission of the People’s Republic of China acknowleged a total of four lists of FMH foods: a total of 86 foods were released in 2002, and additional 9, 6, and 4 foods were supplemented in 2020, 2023, and 2024, respectively, bringing the current list of FMH foods to 105 (Table 2). The clinical application examples of these foods are mainly reflected in the use of prescriptions composed of these foods and other natural extracts (or other forms of drugs) made from these foods. In addition to the examples of prescriptions presented above, the examples or potential uses of FMH food extracts are elaborated in detail in the following sections.

**TABLE 2:** List of FMH substances.

No.	Chinese Name	Latin Name	No.	Chinese Name	Latin Name	No.	Chinese Name	Latin Name
1	Dingxiang	*Eugenia caryophyllata* Thunb.	2	Juemingzi	*Cassia obtusifolia* L. *Cassia tora* L.	3	Luohanguo	*Siraitia grosvenorii* (Swingle.) C. Jeffrey ex A. M. Lu et Z. Y. Zhang
4	Shanzha	*Crataegus pinnatifida* Bge. var. *major* N. E. Br. *Crataegus pinnatifida* Bge.	5	Baihe	*Lilium lancifolium* Thunb. *Lilium brownie* F. E. Brown var. *viridulum* Baker *Lilium pumilum* DC.	6	Yuliren	*Prunus humilis* Bge. *Prunus japonica* Thunb. *Prunus pedunculata* Maxim.
7	Daodou	*Canavalia gladiata* (Jacq.) DC.	8	Roudoukou	*Myristica fragrans* Houtt.	9	Jinyinhua	*Lonicera japonica* Thunb.
10	Xiaohuixiang	*Foeniculum vulgare* Mill.	11	Rougui	*Cinnamomum cassia* Presl	12	Qingguo	*Canarium album* Raeusch.
13	Xiaoji	*Cirsium setosum* (Willd.) MB.	14	Yuganzi	*Phyllanthus emblica* L.	15	Yuxingcao	*Houttuynia cordata* Thunb.
16	Shanyao	*Dioscorea opposita* Thunb.	17	Foshou	*Citrus medica* L. var. *sarcodactylis* Swingle	18	Zhijuzi	*Hovenia dulcis* Thunb.
19	Baizhi	*Angelica dahurica* (Fisch. ex Hoffm.) Benth. et Hook. f. *Angelica dahurica* (Fisch. ex Hoffm.) Benth. et Hook. f. var*. formosana* (Boiss.) Shan et Yuan	20	Tian/Kuxingren	*Prunus armeniaca* L. var. *ansu* Maxim *Prunus sibirica* L. *Prunus mandshurica* (Maxim) Koehne *Prunus armeniaca* L.	21	Sharen	*Amomum villosum* Lour. *Amomum villosum* Lour. var. *xanthioides* T. L. Wu et Senjen *Amomum longiligularg* T. L. Wu
22	Daidaihua	*Citrus aurantium* L. var*. amara* Engl.	23	Yuzhu	*Polygonatum odoratum* (Mill.) Druce	24	Shaji	*Hippophae rhamnoides* L.
25	Machixian	*Portulaca oleracea* L.	26	Huomaren	*Cannabis sativa* L.	27	Qianshi	*Euryale ferox* Salisb.
28	Wumei	*Prunus mume* (Sieb.) Sieb. et Zucc.	29	Pangdahai	*Sterculia lychnophora* Hance	30	Gouqizi	*Lycium barbarum* L.
31	Mugua	*Chaenomeles speciosa* (Sweet) Nakai	32	Fuling	*Poria cocos* (Schw.) Wolf	33	Zhizi	*Gardenia jasminoides* Ellis
34	Huangqi	*Astragalus membranaceus* (Fisch.) Bge. var. *mongholicus* (Bge.) Hsiao *Astragalus membranaceus* (Fisch.) Bge.	35	Dangshen	*Codonopsis pilosula* (Franch.) Nannf. *Codonopsis pilosula* Nannf. var. *modesta* (Nannf.) L.T. Shen *Codonopsis tangshen* Oliv.	36	Muli	*Ostrea gigas* Thunberg *Ostrea talienwhanensis* Crosse *Ostrea rivularis* Gould
37	Chixiaodou	*Vigna umbellata* Ohwi et Ohashi *Vigna angularis* Ohwi et Ohashi	38	Xiangyuan	*Citrus medica* L. *Citrus wilsonii* Tanaka	39	Xiangru	*Mosla chinensis* Maxim. *Mosla chinensis* ‘jiangxiangru’
40	Kunbu	*Laminaria japonica* Aresch. *Ecklonia kurome* Okam.	41	Taoren	*Prunus persica* (L.) Batsch *Prunus davidiana* (Carr.) Franch.	42	Xiebai	*Allium macrostemon* Bge. *Allium chinense* G. Don
43	Baiguo	*Ginkgo biloba* L.	44	Maiya	*Hordeum vulgare* L.	45	Sangshen	*Morus alba* L.
46	Baibiandou	*Dolichos lablab* L.	47	Sangye	*Morus alba* L.	48	Juhong	*Citrus reticulata* Blanco
49	Baibiandouhua	*Dolichos lablab* L.	50	Laifuzi	*Raphanus sativus* L.	51	jiegeng	*Platycodon grandiflorum* (Jacq.) A. DC.
52	Dandouchi	*Glycine max* (L.) Merr.	53	Lianzi	*Nelumbo nucifera* Gaertn.	54	Yizhiren	Alpinia oxyphylla Miq.
55	Juhua	*Chrysanthemum morifolium* Ramat.	56	Gaoliangjiang	*Alpinia officinarum* Hance	57	Heye	*Nelumbo nucifera* Gaertn.
58	Zisu	*Perilla frutescens* (L.) Britt.	59	Danzhuye	*Lophatherum gracile* Brongn.	60	Heizhima	*Sesamum indicum* L.
61	Zisuzi	*Perilla frutescens* (L.) Britt.	62	Gegen	*Pueraria lobata* (Willd.) Ohwi *Pueraria thomsonii* Benth.	63	Heihujiao	*Piper nigrum* L.
64	Fupenzi	*Rubus chingii* Hu	65	Huoxiang	*Pogostemon cablin* (Blanco) Benth. Agastache rugosus (Fisch. et Mey.) O.Ktze.	66	Wushaoshe	*Zaocys dhumnades* (Cantor)
67	Longyanrou (Guiyuan)	*Dimocarpus longan* Lour.	68	Zao (Dazao, Heizao)	*Ziziphus jujuba* Mill.	69	Fengmi	*Apis cerana* Fabricius *Apis mellifera* Linnaeus
70	Feizi	*Torreya grandis* Fort.	71	Ejiao	*Equus asinus* L.	72	Jineijin	*Gallus gallus domesticus* Brisson
73	Suanzao, Suanzaoren	*Ziziphus jujuba* Mill. var*. spinosa* (Bunge) Hu ex H. F. Chou	74	Xianbaimaogen	*Imperata cylindrical* Beauv. var. *major* (Nees) C. E. Hubb.	75	Pugongying	*Taraxacum mongolicum* Hand. -Mazz. *Taraxacum borealisinense* Kitam.
76	Xianlugen	*Phragmites communis* Trin.	77	Huangjiezi	*Brassica juncea* (L.) Czern. et Coss	78	Danggui	*Angelica sinensis* (Oliv.) Diels
79	Jupi	*Citrus reticulata* Blanco	80	Yiyiren	*Coix lacryma-jobi* L. var. *mayuen.* (Roman.) Stapf	81	Shannai	*Kaempferia galanga* L.
82	Huaimi, Huaihua	*Sophora japonica* L.	83	Fushe	*Agkistrodon acutus* (Güenther)	84	Xihonghua	*Crocus sativus* L.
85	Caoguo	*Amomum tsao-ko* Crevost et Lemaire	86	Jianghuang	*Curcuma Longa* L.	87	Biba	*Piper longum* L.
88	Tiepishihu	*Dendrobium officinale* Kimura et Migo	89	Shanzhuyu	*Cornus officinalis* Sieb. et Zucc.	90	Duzhongye	*Eucommia ulmoides* Oliv.
91	Xiyangshen	*Panax quinquefolium* L.	92	Tianma	*Gastrodia elata* Bl.	93	Dihuang	*Rehmannia gluti*nosa Libosch.
94	Maidong	*Ophiopogon japonicus* (L.f) Ker-Gawl.	95	Tiandong	*Asparagus cochinchinensis* (Lour.) Merr.	96	Jiang (Shengjiang, Ganjiang)	*Zingiber officinale* Rosc.
97	Bajiaohuixiang	*Illicium verum* Hook.f.	98	Roucongrong (Huangmo)	*Cistanche deserticola* Y. C. Ma	99	Huajuhong	*Citrus grandis* ‘Tomentosa’ *Citrus grandis* (L.) Osbeck
100	Juju	*Cichorium glandulosum* Boiss. et Huet *Cichorium intybus* L.	101	Bohe	*Mentha haplocalyx* Briq.	102	Lingzhi	*Ganoderma lucidum* (Leyss. ex Fr.) Karst. *Ganoderma sinense* Zhao, Xu et Zhang
103	Huangjing	*Polygonatum kingianum* Coll. et Hemsl. *Polygonatum sibiricum* Red. *Polygonatum cyrtonema* Hua	104	Gancao	*Glycyrrhiza uralensis* Fisch. *Glycyrrhiza inflata* Bat. *Glycyrrhiza glabra* L.	105	Huajiao	*Zanthoxylum schinifolium* Sieb. et Zucc. *Zanthoxylum bungeanum* Maxim.

^a^ Data are retrieved from the official website of National Health and Health Commission of the People’s Republic of China (http://www.nhc.gov.cn/sps/s3593/200810/a298f342c464475b923bcdc3c9d0575a.shtml; http://www.nhc.gov.cn/sps/wslgf/201307/d5865a4304684d6caf418afba0e6527a.shtml; http://www.nhc.gov.cn/sps/s7885/202001/b941b6138e93414cb08aed926ca3c631.shtml; http://www.nhc.gov.cn/sps/s7892/202311/f0d6ef3033b54333a882e3d009ff49bf.shtml; http://www.nhc.gov.cn/sps/s3586/202408/5456157376e840e4990f885e82062e81.shtml).

## 3 Pathophysiology of PACS

Although the research of PACS is gradually increasing, further exploration of explicit mechanisms regulating PACS is needed, due to its multi-system and multi-symptom characteristics. Currently, the widely recognized explanations are based on four factors related to immune dysfunction, including viral factor, inflammatory factor, vascular factor, and microbiota factor (Harky et al., 2023; Tziolos et al., 2023). The mechanisms of different symptoms in each system have their uniqueness and similarities. Furthermore, it cannot rule out the possibility that multiple factors coexist and influence each other.

### 3.1 Viral factor

SARS-CoV-2 binds to the ACE2 receptor on the cell surface and then activates the S protein through transmembrane serine protease 2 (TMPRSS2) before entering the cell. The widespread expression of ACE2 in multiple tissues and organs in the body provides the basis for the multi-system symptoms of PACS (Harky et al., 2023; Vernia et al., 2023). The persistent presence and re-attack of SARS-CoV-2 after infection explain the long-term nature of symptoms (Turner et al., 2023). The reactivation of dormant viruses is generally considered a potential cause of the emergence of new symptoms when the immune response is impaired (Hannestad et al., 2023; Turner et al., 2023). For example, the reactivation of Epstein Barr virus (EBV) and human herpesvirus 6 (HHV-6) is associated with the severity of various neurological disorders, such as fatigue, headache, encephalitis, insomnia, and confusion (Hashimoto, 2023; Leng A. et al., 2023; Vojdani et al., 2023).

### 3.2 Inflammatory factor

Excessive immunity caused by the invasion of SARS-CoV-2 can cause cytokine storm, leading to inflammatory damage and functional abnormalities in multiple systems (Vojdani et al., 2023; Song et al., 2023). The degree and duration of inflammation are closely related to the blood brain barrier (BBB) permeability, lymphocyte transport, innate immune cell infiltration, and endothelial cell injury (Galea, 2021). BBB is an important physiological structure that separates the central nervous system (CNS) from the peripheral circulation (inflammatory mediators and immune cells) (Sonar and Lal, 2018; Huang et al., 2021), which protects neurons and sends signals of inflammation and infection to the brain (Galea, 2021). Peripheral inflammation can destroy BBB and cause various CNS symptoms (Varghese et al., 2024). Furthermore, mast cell activation is also involved in excessive inflammatory response, causing mast cell activation syndrome (MCAS), characterized by multi-system allergic symptoms, such as urticaria and gastrointestinal discomfort (Turner et al., 2023).

### 3.3 Vascular factor

The direct effect of SARS-CoV-2 on endothelial cells and the indirect effect of cytokine storm can cause vascular injury and induce prethrombotic state (Santoro et al., 2023; Zhou et al., 2024). The binding of SARS-CoV-2 to ACE2 leads to the downregulation of ACE2, ultimately leading to increased level of angiotensin II (Ang II) and affecting arterial contraction, blood pressure, and the release of cytokines (Zhou et al., 2024). Endothelial cells are important hubs for regulating inflammation and coagulation (Danese et al., 2007; Six et al., 2022), while ACE2 receptor is expressed in vascular endothelium (Guan Q. Y. et al., 2024). The invasion of virus induces the apoptosis of these cells, affect the cytokines, and cause endothelial dysfunction, while the release of inflammatory mediators and reactive oxygen species (ROS) induce the release of coagulation factors, leading to hypercoagulability and aggravating organ insufficiency and thrombosis (Silva Andrade et al., 2021; Six et al., 2022; Turner et al., 2023; Ren and Li, 2024).

### 3.4 Microbiota factor

Both ACE2 and TMPRRSS2 are present in gut neurons and glial cells, which explains the gut susceptibility to SARS-CoV-2 (Ahmed et al., 2023). Viral invasion and cytokine storms can cause the imbalance of gut microbiota and induce the multi-system symptoms through “lung-gut” axis and “brain-gut” axis (Hashimoto, 2023). For example, the vagus nerve is involved in the composition of the digestive nervous system, which provides a basis for explaining the role of gut factor in the occurrence and development of neurological symptoms of PACS (Ahmed et al., 2023). Th upper respiratory tract is an important site of SARS-CoV-2 infection and proliferation, and the imbalance or variations in the microbiota of the upper respiratory tract are also related to the occurrence and development of PACS (Álvarez-Santacruz et al., 2024).

It is worth noting that the explicit mechanisms underlying PACS described in this section have limited assumptions or supporting evidence. Future physiological and pathological studies are needed to verify the findings reviewed in this study.

## 4 PACS and FMH food intervention in various systems

### 4.1 Nervous system

Nervous system symptoms involve cognitive dysfunction, brain fog, anxiety, depression, sleep disorders, headache, fatigue, and olfactory dysfunction (OD).

Persistent neurological symptoms could be related to damage to the CNS and peripheral nervous system (PNS) (Marques et al., 2023). SARS-CoV-2 enters the brain to affect CNS through PNS, including olfactory nerve, trigeminal nerve, and vagus nerve; SARS-CoV-2 can also infiltrate the CNS by affecting the BBB, e.g., infecting endothelial cells to go through the BBB or triggering inflammatory response to damage BBB (Ahmed et al., 2023). It is suggested that the occurrence of headache is related to historical headache, persistent activation of immune system, activation of tribinovascular system, and changes of gray-white matter and functional connectivity (Tana et al., 2022). Gastrodin has been shown to be beneficial for headache and the specific level of Gastrodin can reduce the expression of CGRP-ir (+) neuron, CGRP-mRNA, and pERK1/2 in rat trigeminal ganglion, and the ERK1/2 pathway is involved in the reduction of CGRP upregulation (Luo et al., 2011).

Damage to endothelial cells can affect capillary function and microthrombi formation, leading to dysfunction of microvascular and alveolar gas exchange and causing multi-organ hypoxemia (Gyöngyösi et al., 2023). The hypoxia of brain or nerve cells and the inflammatory response in CNS, which is caused by microthrombus, can explain the occurrence and development of symptoms, such as delirium, attention disorder, memory impairment, cognitive dysfunction, and mental damage (Ahmad et al., 2022; Song et al., 2023). Akyuva et al. have reported that restoratrol can alleviate hypoxia-induced neuronal cell death by regulating TRPM2 channel (Akyuva and Nazıroğlu, 2020). Neuroinflammation is fundamental immune response of CNS and is closely related to the production of inflammatory mediators and glial cells (Chen et al., 2022b). Glial cells, made up half of the cells in the CNS, protect and repair neurons, regulate metabolism and immunity, and maintain the balance and stability of the nervous system (Allen and Lyons, 2018). For example, A1 astrocytes could alter the BBB permeability, while inflammatory mediators play an important role in the regulation of neuropathic pain and neuroinflammation (Li Q. Y. et al., 2017; Chen et al., 2022b). Studies have shown that gastrodin improves the learning and memory ability of rats with neuroinflammation and reverses the effects of lipopolysaccharide (LPS) on the levels of cytokines in the hippocampus, such as TNF-α, IL-1β, and IL-6; the neuroprotective effect of gastrodin on neuroinflammation in rats is also related to the TLR4-NF-kB-NLRP3 pathway, suggesting its potential as an intervention drug for neuroinflammation-induced cognitive dysfunction (Zheng X. et al., 2022). For example, Li et al. performed the network pharmacology on the potential mechanism of Shengjiang in the treatment of neurodegenerative diseases (NDDs) (i.e., chronic neurological disorders associated with cognitive or motor dysfunction), and obtained 5 core targets (HMOX1, AChE, NOS, COMT, and mGluR5) and 10 main metabolites (e.g., rutin, diacetoxy-[8]-gingerdiol, and curcumin) (Li D. Q. et al., 2022). Xiong et al. showed that chlorogenic acid can improve cognitive dysfunction caused by neuroinflammation by acting on seven targets related to TNF signaling pathway, including Akt1, TNF, MMP9, PTGS2, MAPK1, MAPK14, and RELA (Xiong, 2023).

Depression, anxiety, and sleep disorders can be attributed to psychological stress and tension caused by the COVID-19 pandemic (Ahmad et al., 2022). In addition, systemic vasculitis may also be a potential cause of these symptoms (Kubota et al., 2023). Suanzao (*Z. jujuba* Mill. var*. spinosa* (Bunge) Hu ex H. F. Chou), Fuling (*P. cocos* (Schw.) Wolf), and Gancao are the most commonly used sedative and hypnotic botanical drugs (Singh and Zhao, 2017). Fuling extract can promote inhibitory neurotransmission through γ-aminobutyric acid type A (GABA_A_) receptors, thereby improving sleep quality (Kim et al., 2022). Jujuboside A is an active ingredient in Suanzaoren, and the jujubogenin obtained by its hydrolysis has the most compatible binding conformation with GABA_A_, with strong BBB penetration capability (Chen et al., 2008). Curcumin can also alleviate the anxiety behavior of dextran sulfate sodium salt-induced mice, which is attributed to the regulation of gut microbiota and the increased level of phosphatidylcholine in the prefrontal cortex (Zhang F. et al., 2022). Gallic acid is widely found in a variety of FMH food and is used to treat neurological diseases, such as depression, anxiety, neuropathic pain, memory loss, and neuroinflammation (Bhuia et al., 2023). The network pharmacological analysis of Danggui showed that Danggui plays an anti-depressant role through sphingolipid metabolic pathway, and the E-ligustilide in Danggui has high binding activity with PIK3CA and PIK3CD (Gong et al., 2023). Studies showed that the effect of gastrodin on depressive symptoms in single prolonged stress rats was related to the decreased expression of corticotropin releasing factor in paraventricular nucleus of the hypothalamus and central amygdala and the inhibition of neuron synthesis in locus ceruleus (Zhao R. et al., 2023).

The occurrence of fatigue could be related to excessive immunity, depletion of branched chain amino acids and fatty acids, CNS toxicity accumulation caused by damage to olfactory sensory neurons, mitochondrial dysfunction, and oxidative stress (Chen and Lu, 2023; Guan Y. et al., 2024). TCM (i.e., FMH food) contains many types of active ingredients, which can widely regulate fatigue. For example, polysaccharides can improve resistance by promoting glycogen synthesis, reducing metabolites, and increasing hypoxia tolerance; alkaloids can promote glycogen reserves and improve exercise endurance; glycosides can improve exercise endurance, glycogen content, and lactic acid scavenging ability; and polyphenols can reduce the accumulation of free radicals (Zhou and Jiang, 2019). Resveratrol is a common type of FMH food metabolite, which is widely found in grapes, peanuts, and Sangshen (*Morus alba* L.). The potential anti-fatigue targets of resveratrol (i.e., TP53, PIK3R1, AKT1, PIK3CA, and MAPK1) were identified by network pharmacology, with high binding activity with resveratrol, and the animal experiments showed that resveratrol could prolong the exercise tolerance of mice, reduce the levels of uric acid, blood lactic acid, and blood urea nitrogen, and alter the glycogen storage (Ma et al., 2023). Zhang et al. screened six active metabolites related to cancer-related fatigue from Roucongrong (*Cistanche deserticola* Y. C. Ma), and revealed the potential intervention mechanisms involved multiple targets (AKT1, IL-6, VEGFA, CASP3, and JUN) and multiple pathways (AGE-RAGE and HIF-1); the animal experiments showed that the extracts of Roucongrong (i.e., quercetin, beta-sitosterol, arachidonate, suchilactone, yangambin, and marckine) could improve skeletal muscle atrophy in mice, and the cell experiments indicated that these extracts could reduce the content of ROS and the expression of Beclin-1 protein in cells, increase the number of autophagosomes and the expression of HIF-1α and BNIP3L proteins (Zhang S. L. et al., 2023).

Non-neuronal cells (e.g., sustentacular cells) in olfactory epithelium (OE) can express ACE2 and TMPRSS2, and the invasion of SARS-CoV-2 can damage olfactory neurons and cause cytokine storm (Hu et al., 2023), while virus invasion and long-term inflammation cause irreversible damage to the OE (Leng A. et al., 2023). SNCA/α-synuclein (α-syn) is abundantly expressed in brain tissue, and its function is to regulate synaptic vesicle transport and control the release of neurotransmitters in vesicle, which is related to OD caused by Parkinson’s disease (PD) (Morato Torres et al., 2020). SARS-CoV-2 can reshape protein homeostasis and affect the aggregation of α-syn, which can affect the olfactory pathway in OE and olfactory bulb (OB) (Liu et al., 2023; Mancini et al., 2023). TCM is an effective method for the treatment of post-viral OD (Ma et al., 2021). For example, studies have shown that oral administration of piperine can alleviate the OD of Thy 1-SNCA transgenic mice overexpressing human SNCA and downregulate SNCA (Li R. et al., 2022). OD is also related to olfactory ensheathing cells (Zhou et al., 2023). Curcumin can enhance the proliferation of these cells, and increase cell viability and secretion of both neurotrophic factors and anti-inflammatory factors (Guo et al., 2020).

### 4.2 Respiratory system

Lung is the main target organ of SARS-CoV-2 infection, and the respiratory symptom is one of the most common symptoms in PACS patients, including dyspnea, cough, PF, and pulmonary dysfunction (Kanne et al., 2023).

The infection of SARS-CoV-2 cause the overexpression of ACE2 receptor, leading to the imbalance in some physiological systems and the acute inflammatory pulmonary edema and thromboembolism (Silva Andrade et al., 2021). The formation of pulmonary thrombosis blocks the pulmonary vessels, causing the dysfunction of pulmonary circulation and respiratory, resulting in hypoxia and dyspnea (Song et al., 2023). Patients with COVID-19 combined with acute or chronic respiratory diseases often experience cough symptoms (Watase et al., 2023). This cough, which lasts for weeks or months, is different from other types of cough caused by conventional influenza virus, rhinovirus, and RS virus infections, and is related to various organ symptoms, such as difficulty breathing, cognitive impairment, and fatigue (Song et al., 2021; Watase et al., 2023). This type of cough may be associated with a state of cough hypersensitivity induced by SARS-CoV-2, involving neurotropism, neuroinflammation, and neuroimmunomodulation (Song et al., 2021). As described above, the effects of several botanical drugs/metabolites on neuroinflammation are probably the potential mechanisms intervening cough by FMH food. In TCM, it is believed that the pathogenesis of cough is often attributed to either *Fei Shi Xuan Jiang* (i.e., impaired diffusion and downbearing of the lung or in brief, pulmonary dysfunction) or *Zang Fu Gong Neng Shi Tiao* (i.e., disorder of organ functions) (Zhang C. K. et al., 2023). Furthermore, this type of cough caused by SARS-CoV-2 has important diagnostic and therapeutic values in both the acute and late stages of SARS-CoV-2 (Zheng H. et al., 2023). Liquiritin in Gancao, platycodin D in Jiegeng, and amygdalin in Xingren have been demonstrated to have antitussive effects (Miyagoshi et al., 1986; Li Q. et al., 2022; Qin et al., 2022). The anti-inflammatory and antitussive effects of liquiritin are mediated by the dual inhibition of TRPV1 and TRPA1 channels, and during airway inflammation, both effects are upregulated through the NF-κB pathway in non-neuronal cells (Liu Z. et al., 2020). The fermented Jiegeng extract caused increased level of platycodin D, which significantly reduced both the number of coughs and the cough reflex sensitivity, showing evident anti-inflammatory effects, as demonstrated by the variations in the level of TNF-α, IL-6, and IL-1β (Lee et al., 2020). Amygdalin inhibits airway epithelium apoptosis, inflammation, and epithelial-mesenchymal transition by regulating TLR4/NF-κB pathway, thereby interfering with the onset and progression of cough-variant asthma (i.e., characterized primarily by coughing) (Si and Zhang, 2021). In TCM, products of plants in the genus *Lilium* are commonly used in the treatment of cough and lung diseases (Zhou et al., 2021). For example, Hu et al. investigated Baihe (*Lilium lancifolium* Thunb.) products to show that freeze-drying Baihe, drying Baihe, and fresh Baihe could improve cough sensitivity, change the serum levels of IFN-γ, IL-4, and IL-8, downregulate the expression of SP, IL-5, and TNF-α, and improve lung function (Hu, 2022). In addition, PF is also associated with cough and breathing difficulties (Martinez et al., 2017).

SARS-CoV-2 diffuses into the respiratory system through nasal epithelial cells, binds to ACE-2 receptors on lung epithelium, infects type II alveolar cells in the lower respiratory tract, ultimately causing diffuse alveolar damage (DAD) (Mohammadi et al., 2022; Pi et al., 2023). Lung injury is often accompanied by inflammation, which activates inflammatory mediators, promotes cell recruitment and release of profibrotic mediators, and further activates pulmonary fibroblasts into myofibroblasts. Abnormal and constant activation of myofibroblasts causes secretion of extracellular matrix (ECM) metabolites, leading to excessive ECM deposition and PF formation (Pi et al., 2023). PF is generally a long-term complication in severe patients (Sideratou and Papaneophytou, 2023). TGF-β1 is one of core targets of PF, playing a positive role in the development of idiopathic pulmonary fibrosis (IPF) through many signaling pathways, such as Smad, MAPK, and ERK (Ye and Hu, 2021). Resveratrol, curcumin, and gallic acid are the potential drugs for the intervention of PF (Wang L. C. et al., 2022). Quercetin is detected in many botanical drugs and has been used to improve bleomycin (BLM)-induced PF, alter the expression levels of hydroxyproline, fibronectin, α-smooth muscle actin, Collagen I, and Collagen III, and inhibit SphK1/S1P signal transduction (Zhang et al., 2018). The network pharmacology analysis showed that quercetin is involved in mediating cancer-related pathways, PI3K-Akt pathway, and Ras pathway to interfere with IPF by acting on AKT1 and PIK3CG (Zhang et al., 2021). Studies have shown that hesperidin, which is detected in Juhua (*Chrysanthemum morifolium* Ramat.), could downregulate the expression levels of senescence marker proteins (e.g., p53, p21, and p16), inhibit the IL6/STAT3, TGF-β1/Smad3/AMPK, and IκBα/NF-κB pathways, regulate the levels of TNF-α, IL-1β, and IL-6, and alleviate PF in BLM-induced mouse model (Zhou et al., 2019; Han et al., 2023). Platycodin D can downregulate TRPC6, reduce ROS production, and alleviate PF in mice (Liang et al., 2024). Houttuynin is one of the main metabolites detected in Yuxingcao (*Houttuynia cordata* Thunb.), and studies have shown that sodium houttuyfonate (i.e., sodium bisulfite and houttuynin) with high stablity have shown therapeutic effects in relieving PF (Shen et al., 2021; Zhu et al., 2021). The network pharmacology revealed the potential mechanism of Zhebeimu (*Fritillaria thunbergii* Miq.) against IPF, based on a total of 86 active ingredients, such as pelargonidin and kaempferol, ands 9 potential targets, with the results verified by molecular docking and qRT-PCR analyses (Xu G. et al., 2022). These studies suggest that both pelargonidin detected in Shaji (*Hippophae rhamnoides* L.) and kaempferol found in many FMH foods could provide potential clinical treatment for IPF. Huangqi (*Astragalus membranaceus* (Fisch.) Bge. var. *mongholicus* (Bge.) Hsiao) is effective for many fibrotic diseases, capable of inhibiting epithelial-mesenchymal transition (EMT), ROS, and TGF-β1/Smads (Zhu et al., 2022). Total flavonoids extracted from Huangqi are demonstrated to regulate EMT through TGF-β1/Smad pathway and enhance the expression of Wnt7b protein, which is involved in alveolar epithelium repair (Yang C. G. et al., 2023). Studies have shown that astragaloside IV could inhibit TGF-β1/PI3K/Akt-induced hyperphosphorylation and downregulation of FOXO3a, prevent PF by inhibiting TGF-β1/Smad pathway, downregulate the expression of Collagen I, fibronectin, and α-SMA, and reduce inflammatory response and oxidative stress (Qian et al., 2018; Li et al., 2021b). Furthermore, the anti-PF effect of astragaloside IV is also involved in RAS/RAF/FoxO pathway and lncRNA-ATB/miR-200c/ZEB1 pathway (Zhang M. et al., 2023; Guan Q. Y. et al., 2024).

It is worth noting that IPF is a typical example of chronic progressive fibrosis, similar to PACS-related PF, with its exacerbation caused by SARS-CoV-2 infection (Patrucco et al., 2023; Yang C. et al., 2024). PACS-related PF could be also caused by pneumonia induced by either acute respiratory distress syndrome (ARDS) or COVID-19, thus the patients could benefit from the treatment of IPF (Duong-Quy et al., 2023).

### 4.3 Cardiovascular system

The symptoms of PACS patients in the cardiovascular system include chest pain, postural orthostatic tachycardia syndrome (POTS), palpitation, hypertension, and myocardial injury (Philip et al., 2023), and both palpitation and chest pain are the most common symptoms (Sideratou and Papaneophytou, 2023).

PACS related cardiovascular symptoms are closely associated with cellular dysregulation (e.g., interferon-γ-screening T cells and specific memory B cells), mitochondrial dysfunction and oxidative stress, vascular and endothelial dysfunction, and thrombotic complications (Gyöngyösi et al., 2023). Furthermore, SARS-CoV-2 could directly infect heart cells, causing viral myocarditis and leading to abnormal heart functions (Ren and Li, 2024). Myocarditis is a complication of COVID-19, occurring in patients of all ages, and closely related to the direct viral damage and excessive host immune response (Li et al., 2021c). Curcumin is used to protect myocardial cell injury after ischemia and hypoxia (Yang S. S. et al., 2024), exerting cardioprotective effects by up-regulating DKK-3 pathway and inhibiting p38 and JNK pathways (Cao et al., 2018). Huangqi is commonly used to dilate blood vessels, lower blood pressure, reduce the risk of atherosclerosis, and improve cardiac function (Li J. J. et al., 2024). Both formononetin and calycosin are two isoflavones detected in Huangqi. Calycosin is used to alleviate myocardial fibrosis and cardiac dysfunction *in vivo* and inhibit TGF-β1-induced CFs proliferation and collagen deposition by inhibiting TGFBR1 pathway *in vitro* (Chen G. et al., 2022), while formononetin is demonstrated to reverse the decrease of *ALDH2* mRNA and *HADH* mRNA expression and the increase of MOAB in cardiomyocytes after ISO treatment, improve mitochondrial dysfunction, and alleviate cardiac fibrosis (Qian et al., 2024). Astragaloside IV has been shown to benefit mitochondrial bioenergetics of cardiomyocytes (Huang et al., 2018), protecting the heart by regulating the expression of Nrf-2, HO-1, HIF-1α, JAK2/STAT3, TLR4, MyD88, and NF-κB p65 (Zang et al., 2020; Li B. et al., 2021; Shi et al., 2021; Chen et al., 2023). JAK2/STAT3 signaling pathway plays an important role in a variety of inflammatory diseases. For example, studies have shown that promoting the activation of JAK2/STAT3 signaling pathway benefits myocarditis caused by viral infection (Cai et al., 2023). The specific roles and mechanisms of Nrf-2, HO-1, HIF-1α, TLR4, MyD88, and NF-κB are further discussed below in Section 5.

Hypertension is suspected to be a potential symptom of PACS, i.e., some patients are diagnosed with newly developed arterial hypertension after being infected with COVID-19 (Tobler et al., 2022; Wrona and Skrypnik, 2022). In TCM, flavonoids have been shown to be beneficial for cardiovascular system, affecting blood pressure via various ways (Cao et al., 2021). Gastrodin has been demonstrated to improve myocardial hypertrophy, hypertension, and myocardial ischemia-reperfusion injury (Xiao et al., 2023). Ethyl cinnamate, an active ingredient in rhizomes of Gaoliangjiang (*Alpinia officinarum* Hance), exerts vasorelaxation activity (Othman et al., 2002). Huaihua (*Sophora japonica* L.) has also been reported to have the antihypertension effect (He et al., 2016). Puerarin is used to reduce diastolic blood pressure and heart rate in spontaneously hypertensive rats, enhance the levels of NO and cGMP and the expression of phosphorylated eNOS protein, and reduce the levels of AT1 and Cav1 (Shi et al., 2019). Gouqizi could inhibit the expression of PI3K and Akt proteins induced by Hcy and increase the expression of miR-145 (Zhang M. et al., 2020). Anthocyanin isomers in *Lycium ruthenicum* Murray (Pt3R5G) can inhibit ACE and could be served as potential plant-derived ACE inhibitors (Lu et al., 2023). Yiyiren (*Coix lacryma-jobi* L. var. *mayuen.* (Roman.) Stapf) seeds are rich in storage proteins; in particular, hydrolyzed bioactive oligopeptides have been shown to be effective for the treatment of hypertension (Qiao et al., 2016).

It is well known that long-term inflammation could cause cardiac remodeling due to fibrosis, ultimately leading to heart failure or arrhythmia (Korompoki et al., 2021). COVID-19 affects the epicardial nerves, manifested as inflammatory neuropathy, leading to cardiac complications such as myocardial injury and arrhythmia (Weissert, 2024; Zhou et al., 2024). SARS-CoV-2-mediated or immune-mediated autonomic nervous system damage could also lead to orthostatic intolerance, dyspnea, chest pain, palpitation, and other symptoms (Dani et al., 2021). Cav1.2 plays an important role in hypertension, atherosclerosis, heart failure, and other diseases (Loh et al., 2023). Uncarialin A is an alkaloid detected in Pugongying (*Taraxacum mongolicum* Hand. -Mazz.), capable of inhibiting L-type calcium channel subunit alpha-1C (Cav1.2) through hydrogen bond interaction with amino acid residue Met1186, thereby inhibiting Ca^2+^ inward current (Yun et al., 2020). Arrhythmia is associated with cardiac electrophysiological disorders, and studies have shown that quercetin mitigates arrhythmia by modulating Na^2+^ channel, K^+^ channel, and Ca^2+^ channel (Zhou Y. et al., 2022). Furthermore, curcumin acts on K_to_ channel, puerarin acts on K_1_ and K_s_ channels (Wang C. et al., 2023), puerarin, glycyrrhetinic acid, and hesperetin affect Na^2+^ channel (Zheng et al., 2021), and procyanidins, resveratrol, 6-gingerol, and 8-gingerol modulate Ca^2+^ channel (Zhang X. et al., 2023).

### 4.4 Digestive system

The common symptoms of PACS patients in the digestive system include irritable bowel syndrome (IBS), inappetence, nausea, vomiting, abdominal pain, and diarrhea.

The enteric nervous system contains a large number of neurons and neurotransmitters, and the autonomic nerve supply in the gastrointestinal tract is influenced by many factors, such as emotion, drug, and hormone (Elbeltagi et al., 2023). The release of neuroactive agents and inflammatory mediations in the gut affects the abdominal vagal nerve and stimulates the dorsal medulla nerve, causing nausea and vomiting (Elbeltagi et al., 2023). Acute and delayed vomiting is associated with the interaction between 5-HT3 and NK1 receptor neurotransmitter pathways (Rojas and Slusher, 2012). Studies have shown that Shengjiang extracts accelerate gastric emptying and stimulate gastric antral contractions (Giacosa et al., 2015). The 6-gingerol, 8-gingerol, 10-gingerol, and 6-shogaol in Shengjiang are used as antagonists of 5-HT3 and NK1 (Pertz et al., 2011; Haniadka et al., 2012). Anticancer drugs, such as cisplatin, could cause nausea and vomiting, the expression of kaolin intake and Fos is considered an evaluation index (Horn, 2009). Studies have shown that Lingzhi (*Ganoderma lucidum* (Leyss. ex Fr.) Karst.) polysaccharides are used to reduce kaolin intake and Fos expression in mice and alleviate cisplatin-induced vomiting (Yan et al., 2009). Xiyangshen (*Panax quinquefolium L.*) berry extract and one of its components, ginsenoside Re, have shown similar effect (Mehendale et al., 2005), while Huoxiang (*Pogostemon cablin (Blanco) Benth.*) is also used to treat nausea and vomiting (Xu F. et al., 2022).

Anorexia, a common symptom during the infection and recovery period of COVID-19, also occurs in PACS patients. Poor communication between the brain and gut could cause changes in brain 5-HT and tryptophan concentrations, leading to anorexia and OD (Elbeltagi et al., 2023). Appetite is regulate by targets such as cholecystokinin (CCK) and glucagon-like peptide-1 (GLP-1), while the Danggui aqueous decoction is shown to influence CCK-8, and the proanthocyanidins affect GLP-1 expression (Feng, 2022). CCK, gastrin, and secretin can affect the parasympathetic nervous system and stimulate digestion (Elbeltagi et al., 2023). Currently, research on appetite intervention is mainly focused on controlling appetite and weight loss. In the future, studies are necessary to identify food ingredients that stimulate appetite.

In addition to various symptoms caused by SARS-CoV-2 infection, the gastrointestinal symptoms caused by COVID-19 are also closely associated with the imbalance of gut microbiota, which could also influence other systems through various “axes” (Gang et al., 2022; Ancona et al., 2023). Gut microbiota is an important target for regulating and improving gut function, which has received extensive attention in recent years. The brain-gut axis is refered to the relationship between the microbiome in the gastrointestinal tract and the communication with the brain through circulating metabolites (Duve et al., 2024). SARS-CoV-2 infects intestinal epithelial cells and causes inflammation, triggering neuroimmune interactions, activating sensory neurons, and affecting related functions through neural pathways (Scheithauer et al., 2024; Verma et al., 2024). SARS-CoV-2 infection reduces the number of butyrate-producing bacteria and leads to excessive induction of inflammation, while the persistence of virus and the change of gut microbiota could destroy the “brain-gut axis,” preventing the communication between CNS and the gut system (Vakili et al., 2022; Ashktorab et al., 2024). Gut and airway dysbiosis are involved in COVID-19 and PACS-related neurological symptoms (Ancona et al., 2023). Respiratory system and gastrointestinal system are derived from the same embryonic organ (foregut), and their microbiota are closely related, suggesting the important role that the “lung-gut axis” plays (Paramythiotis et al., 2023). The “gut-lung axis” is refered to the two-way interaction between the respiratory mucosa and the gut microbiota (de Oliveira et al., 2021). Gut microbiota imbalance is involved in excessive inflammation and multiple organ failure through communication with multiple organs (Chen and Vitetta, 2022). Since the respiratory tract is the main entry point for SARS-CoV-2 infection, the “gut-lung axis” could be the earliest axis affected by COVID-19. Botanical drugs have a long history of treating lung diseases through the mechanisms involving the gut (He et al., 2022). The theory of “lung and large intestine being exteriorly-interiorly related” in TCM is consistent with the theory of “lung-gut axis” in modern medicine, suggesting that targeted therapy for gut microbiota and fecal transplantation could contribute to the treatment of COVID-19 and the relief of gut symptoms in PACS (Hong et al., 2022). Polysaccharides of FMH foods play an important role in intestinal mucosal protection, inflammatory regulation, gut microbiota regulation, and the recovery of gut function (Li X. et al., 2023). Flavonoid, the main metabolite of Gancao, has been shown to increase the activity of gastric mucosal epithelial cells of gastric ulcer rats, restore the gastric mucosal barrier, alleviate the disorder of gut microbiota, increase the concentration of short-chain fatty acids (SCFAs), and play a positive role in gastric mucosa by activating EGFR/ERK pathway (Wu et al., 2023). Polysaccharides from *Lycium barbarum* fruits can reduce gut injury, and their effects on certain metabolic pathways oppose to those of LPS, suggesting their potential as regulators of gut function (Yan et al., 2022). Flavonoids, polysaccharides, and saponins of Huangqi have been shown to maintain gut homeostasis by affecting the composition and metabolism of gut microbiota (Tian et al., 2020). Fupenzi (*Rubus chingii* Hu) polysaccharides are used to improve HFD-induced gut microbiota imbalance, produce SCFAs, and maintain gut barrier integrity (Huang et al., 2024). Additionally, the polysaccharides of Shanyao, Dangshen (*Codonopsispilosula* (Franch.) Nannf.), and Fuling have also been shown to improve gut inflammation by regulating the composition and function of gut microbiota (Song et al., 2022; Yang X. et al., 2023).

IBS is a chronic intestinal disease that can lead to changes in abdominal pain and bowel habits, including IBS with constipation (IBS-C), IBS with diarrhea (IBS-D), IBS with a mixed pattern of constipation and diarrhea, and unclassified IBS, thus controlling these symptoms is the focus of treatment of IBS (Bahrami et al., 2016; Bonetto et al., 2021; Liu et al., 2024). IBS is one of the most common disorders involving gut–brain interaction, which is affected by multiple factors, such as virus infection, gut inflammation, HPA axis, and Dietary change (Paramythiotis et al., 2023). Studies have shown that curcumin is used to treat IBS-D by regulating gut microbiota and reducing serum levels of HT, SP, and VIP (Tu et al., 2023). The network pharmacology study on the effect of Danggui Shengjiang Mutton Decoction (containing Danggui, Shengjiang, and mutton) has showed that the decoction acts on multiple targets through multi-metabolites, enhances the intestinal mucosal barrier, and regulates intestinal peristalsis (Liu et al., 2024). Yang et al. identified a polysaccharide with β-isomerized configuration and pyranose form from Fuling, showing characteristics beneficial for IBS patients, including water-swelling and water/oil-holding capacity, fructose adsorption capacity, and modulation of IBS-related gut microbes, such as *Lachnospiraceae* and *Prevotella* (Yang X. et al., 2023). Rougui (*Cinnamomum cassia* Presl) is used to prevent IBS-D, showing that the extract of Rougui (containing procyanidin B1/B2, catechin, cinnamic acid, cinnamic aldehyde, and cinnamyl alcohol) can reduce the frequency of defecation of MS rats in a dose-dependent manner, relieving symptoms by regulating the expression of Tph1 and the synthesis of 5-HT (Yu et al., 2022). In TCM, Gegen (*Pueraria lobata* (Willd.) Ohwi) is used to treat diarrhea due to “spleen deficiency,” while puerarin, the main active ingredient of Gegen, can regulate the expression of CRF1, p-ERK/ERK, and sealin, enhance the proliferation of colonic epithelial cells, repair the colonic mucus barrier, and improve the abdominal pain and diarrhea caused by IBS-D (Wang et al., 2021). Yuxingcao aqueous extract causes enhanced expression of ZO-1, occludin, and L-10 and reduced levels of IL-1β, IL-6, and epidermal growth factor receptor (EGFR), playing a positive role in the gut function (Wang X. et al., 2022).

### 4.5 Urogenital system

The symptoms of PACS patients in the urogenital system include renal function decline, sexual dysfunction, and decreased sperm count.

Inflammation, hypercoagulability, microvascular thrombosis, endothelial dysfunction, and mitochondrial dysfunction can induce acute kidney injury (AKI), which could further develop into chronic kidney disease (CKD) under chronic inflammation and cytokine storm (Rai, 2023). The morphological damage in kidney injury mainly occurs in the kidney tubules, with the proximal tubular cells (PTC) severely damaged (Garrett et al., 2023; Rai, 2023). ACE2 receptor is highly expressed in PTC, while the invasion of SARS-CoV-2 could lead to proximal tubule dysfunction (Werion et al., 2020). Creatine phosphate metabolism, un-reclaimed glomerular filtrate, and PTC injury may be associated with PACS (Garrett et al., 2023). In brief, PACS may cause kidney problems (such as renal tubular dysfunction, impaired metabolism, and filtration function), which are mainly associated with SARS-CoV-2 receptor expression, inflammation, abnormal blood circulation, and hormone imbalance. To date, many FMH food ingredients have been demonstrated to be effective against AKI, such as curcumin, quercetin, resveratrol, dioscin, and glycyrrhizin (Yin et al., 2016).

Androgen receptors are associated with SARS-CoV-2 infection, thus male reproductive organs are affected by SARS-CoV-2 infection, and endothelial dysfunction, testicular injury, and psychological burden may lead to erectile dysfunction (ED) (Kaynar et al., 2022). The decrease of serum levels of LH and testosterone indicates the hypothalamic-pituitary-gonadal axis dysfunction, which is closely associated with PACS-induced ED (Al-Kuraishy et al., 2023). SARS-CoV-2 infection also upregulates the expression of pro-inflammatory cytokines, resulting in the destruction of blood-testis barrier and impairment of spermatogenesis (Leng T. et al., 2023). Additionally, changes in ACE2 signaling pathway and oxidative stress could cause abnormal sperm motility and male infertility (Haghpanah et al., 2021).

Studies have shown that the active ingredients of Xiyangshen, Gouqizi, and Shengjiang could reduce the level of ROS, increase sperm motility, and improve conception rate (Wang et al., 2018). Gouqizi polysaccharides improve the levels of IL-2, IL-12, and TNF-α in mice with impaired reproductive system, playing a positive role in the regulation of sperm density and sperm motility (Qian, 2019). For example, a dose of 20 mg/kg Gouqizi polysaccharides reduced testicular spermatogenic damage in mice and regulated the expression of GPX4 and AIF (Liu et al., 2021), while a lower dose of 10 mg/kg improved the sperm quality, the expression of TNP2 and RPL31, and the apoptosis of germ cells (Liu C. et al., 2020). In addition, Gouqizi polysaccharides also reduced the levels of ROS and malondialdehyde (MDA), and increased the activities of superoxide dismutase and glutathione peroxidase (Zhou J. et al., 2022). Xiyangshen is rich in ginsenosides, which are beneficial for improving reproductive function, and the combination of cyclophosphamide and Xiyangshen enhanced the levels of sperm-related indicators (Akram et al., 2012; Hosseini et al., 2018). Ginsenoside Rg1 can activate Akt/BAD signal transduction and reduce D-gal-induced apoptosis of spermatogonia (Wang Z. et al., 2023), while Ginsenoside Re is also beneficial for sperms through the NO/cGMP/PKG pathway (Zhang et al., 2007).

Luteinizing hormone plays a crucial role in animal estrus by stimulating the development of Leydig cells and testosterone secretion, as well as stimulating sperm maturation; testosterone is an androgen produced by the testis and involved in the development of secondary sexual characteristics and the regulation of spermatogenesis (You et al., 2022). In TCM, Shengjiang can affect testosterone and luteinizing hormone levels, improving semen quality by enhancing sperm concentration and motility (Banihani, 2019). Both phenolic acids and flavonoids in Shengjiang can improve the redox state and proliferation ability of testis (Khwanes et al., 2022). Furthermore, the combination of Shengjiang and Rougui has also been shown to be beneficial for sperm motility, serum total testosterone level, and serum antioxidant level, particularly for the reproductive function of diabetic male patients (Khaki et al., 2014). For example, Bordbar et al. (Bordbar et al., 2013) have reported that at a dose of 100 mg/kg, Shengjiang ethanol extract could improve sperm parameters, testosterone level, and the volumes of the testes and seminiferous tubules in rats. In addition, Shengjiang can also enhance the antioxidant capacity (i.e., tHcy and MDA) and hormone levels in rat testis tissue (Motawee et al., 2022; Akbari et al., 2017).

Reproductive problems of women are mainly involved in menstrual cycle, menopause, gonads, ovarian function, and fertility disorders. In severe cases, infertility and abortion symptoms may occur (Pollack et al., 2023; Pourmasumi et al., 2023). These problems are associated with a series of immune responses induced by the binding of ACE2 receptor, which is widely present in the reproductive organs (such as ovary, uterus, vagina, and placenta), while the processes of follicular development, ovulation, angiogenesis are regulated by ACE2 receptor and angiotensin (Dong et al., 2024). Meanwhile, the infection of SARS-CoV-2 could also lead to the damage in the number of blastocysts, top-quality embryos, and euploid embryos (Carp-Veliscu et al., 2022). Studies have shown that botanical drugs play an important role in regulating reproductive hormone disorders, reducing ovarian inflammatory damage, oxidative stress, apoptosis, and follicular atresia, while also improving ovarian pathological damage and reserve function (Siyu et al., 2024). Many metabolites are involved in these processes. For example, quercetin can increase the levels of estradiol and anti-Müllerian hormone as well as progesterone, reduce the levels of follicle-stimulating hormone and luteinizing hormone, and improve ovarian function through the regulation of PI3K/Akt/FoxO3a signaling pathway (Chen et al., 2022c; Zheng S. et al., 2022). Similarly, Gouqi polysaccharides can also improve the hormone levels and reduce pathological changes in ovarian tissue (Zheng W. B. et al., 2023).

### 4.6 Other systems

In addition to the above symptoms, PACS patients may also have rash, hair loss, and other symptoms. In post stage of COVID-19, two types of alopecia have attracted attention, i.e., telogen effluvium (diffuse alopecia mediated by growth cycle disorders) and alopecia areata (patchy, non-diffuse alopecia mediated by immunity and inflammation) (Popescu et al., 2022). The cause of hair loss may be caused by the psychological stress induced by the COVID-19 pandemic, while the effects of hydroxychloroquine, azithromycin, and other drugs cannot be ruled out (Olds et al., 2021; Wise, 2022). Furthermore, the direct effects of pro-inflammatory cytokines, viruses on hair follicles, and microthrombus are also considered responsible for the hair loss process (Inamadar, 2022). Androgenic alopecia and alopecia areata are the two most common forms of hair loss (Zgonc Škulj et al., 2020). However, it is worth noting that androgenic alopecia is considered a marker of COVID-19 severity of, but not of PACS (Popescu et al., 2022). Studies have shown that the patients with alopecia areata eating Shengjiang could significantly improve the oxidation/antioxidant balance of red blood cells and lymphocytes and restore normal serum level of zinc (Abbas, 2020). The network pharmacology analysis showed that 6-Methylgingediacetate, 10-Gingediol, gingerenone B in Shengjiang volatile oil act on TNF, IL6, and ALB to improve the alopecia by regulating both inflammation and immune pathways (Pan et al., 2022). However, these results are based solely on virtual analyses of the metabolites of Shengjiang. Quercetin is widely present in FMH foods, and its local delivery can stimulate the growth of resting hair follicles and affect the microvascular system around hair follicles (Zhao Q. et al., 2023). Quercetin-loaded nanoemulsions and microneedle patches have been developed to promote hair regeneration by alleviating oxidative stress, promoting angiogenesis, and inhibiting dihydrotestosterone and inflammation (Dario et al., 2020; Zhang Z. et al., 2023; Chen et al., 2025). Xiebai (*Allium macrostemon* Bge.) extract (containing adenosine, guanosine, L-phenylalanine, macrostemonoside J, macrostemonoside F, and macrostemonoside O) has been shown to promote the proliferation of HDPC cells in androgenetic alopecia mice, and involve in the activation of Wnt/β-catenin and the production of hair follicle growth factor (Gao et al., 2023). Additionally, Tiepishihu (*Dendrobium officinale* Kimura et Migo) polysaccharides are demonstrated to improve testosterone-induced alopecia and alter Wnt signaling and hair follicle stem cell function (Zhang Y. et al., 2023).

**FIGURE 1:**
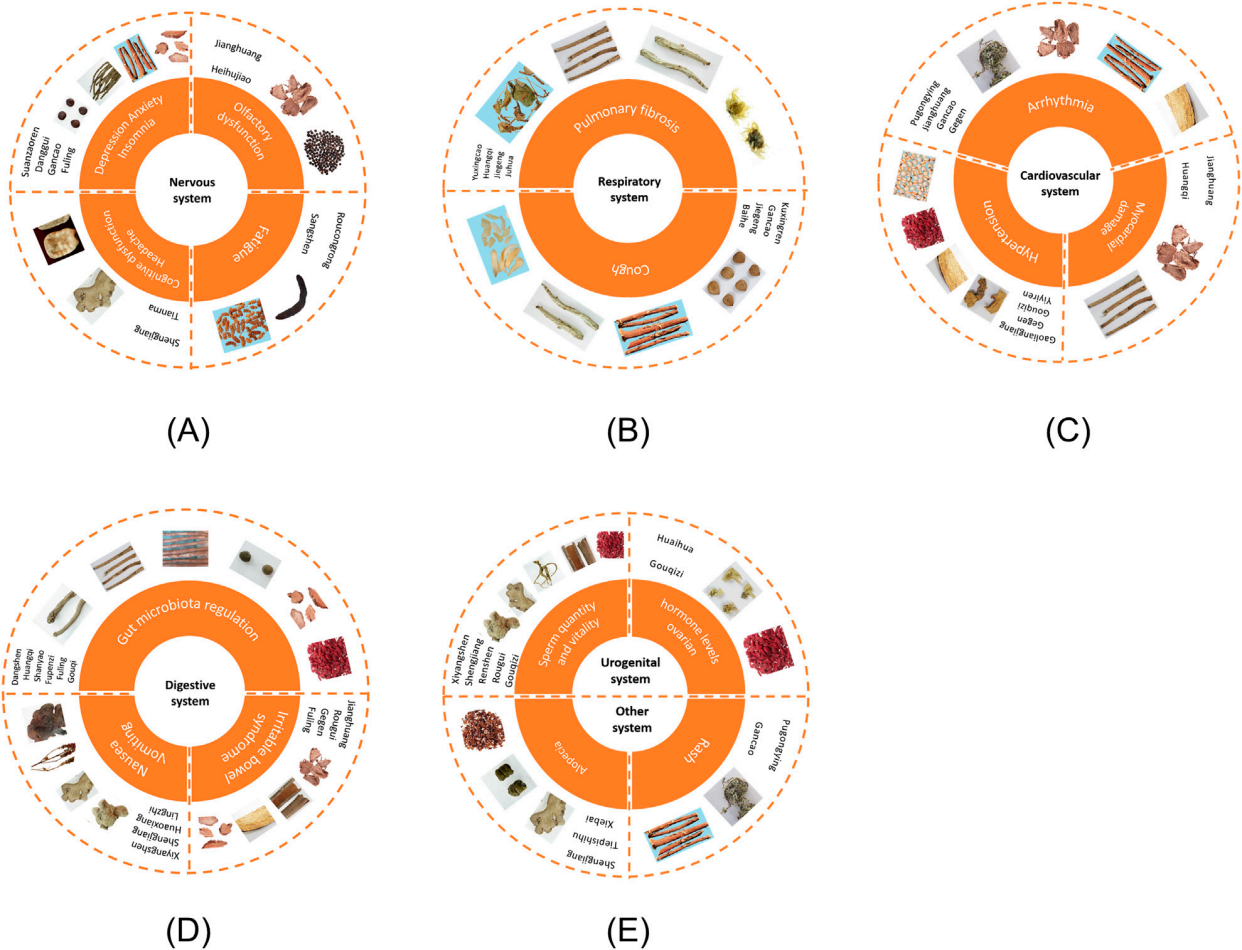
FMH foods related to symptom intervention of PACS in various systems, including **(A)** nervous system; **(B)** respiratory system; **(C)** cardiovascular system; **(D)** digestive system; **(E)** urogenital system and other systems. The material images are retrieved from Chinese medicine specimen center of Nanjing University of Chinese Medicine (https://zybb.njucm.edu.cn/index.asp) and Chinese Medicinal Material Images database (https://library.hkbu.edu.hk/electronic/libdbs/mmd/index.html).

**FIGURE 2:**
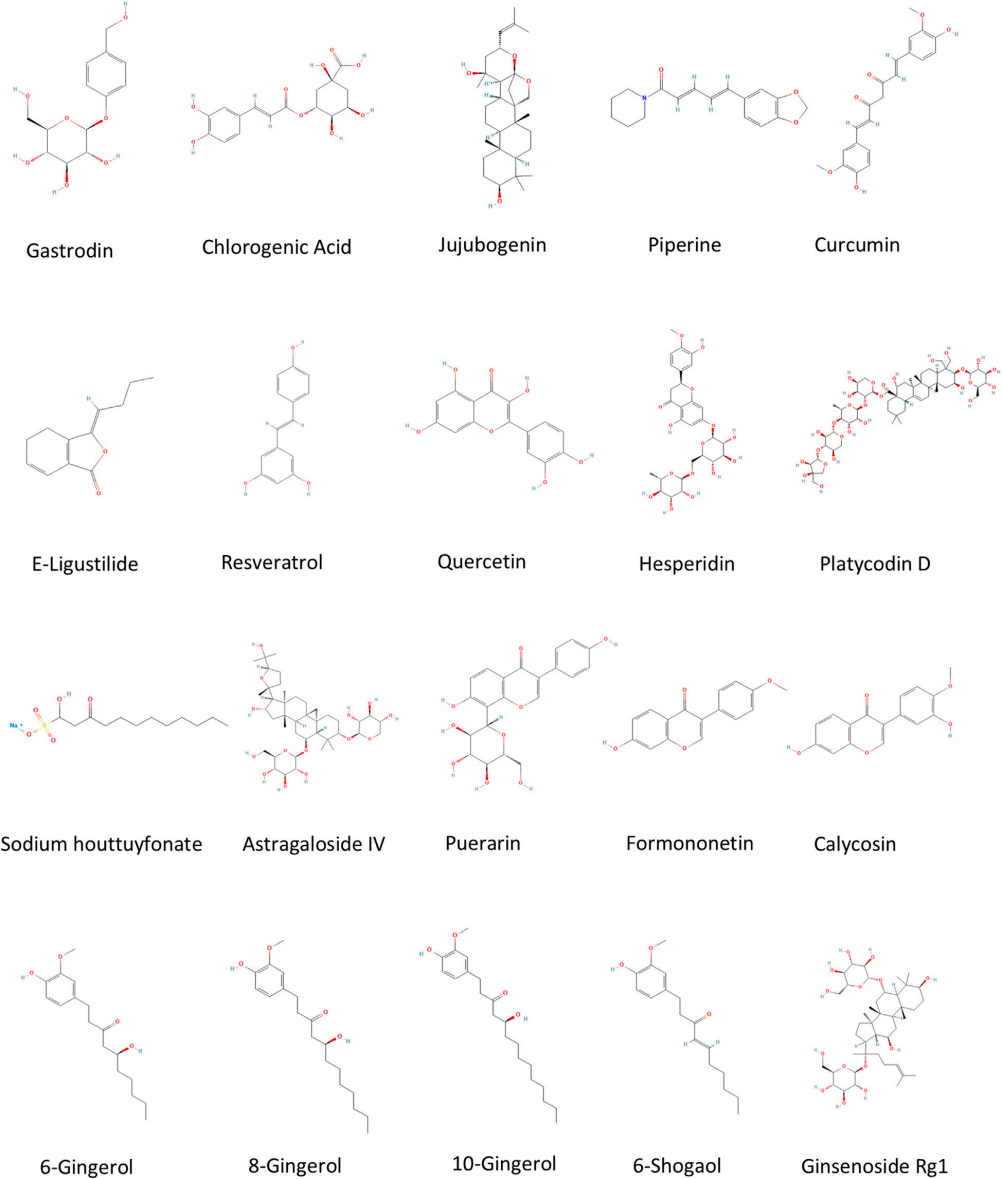
Representative chemical structure diagrams of FMH food derived from Pubchem database (https://pubchem.ncbi.nlm.nih.gov/).

Skin diseases caused by COVID-19 are related to the inflammatory response of spike protein, vitamin D deficiency, activation of ACE2 receptor, androgen level, and psychological stress (Pendlebury et al., 2022). Prtajin et al. reported that the occurrence of pityriasis rosea may be related either to the direct triggering of SARS-CoV-2 or to the indirect triggering caused by the reactivation of other viruses, such as HHV-6 or HHV-7 (Prtajin and Ljubojević Hadžavdić, 2022). Gancao and its active ingredients have been demonstrated to be effective for the treatment of skin diseases, such as eczema, pityriasis rosea, urticaria, and systemic lupus erythematosus, playing a signficant role in inflammatory and allergic reactions and skin tumors (Zhang et al., 2013). The anti-inflammatory experiments based on Pugongying extract (10% flavonoid) revealed that the flavonoid reduced the levels of IL-6 and IL-8, and showing a sound anti-inflammatory effect on the skin (Zhang L. X. et al., 2023). The FMH foods are involved in this section are demonstrated in Figure 1, metabolites are demonstrated in Figure 2 and mechanisms are demonstrated in Table 3.

**TABLE 3:** Summary of FMH food metabolites on PACS intervention^a^.

Name	Source	Molecular weight (g/mol)	Molecular formula	PubChem CID	Efficacy	Dose	Model	Relevant Target/Indicator	Reference
Gastrodin	Tianma/*Gastrodia elata* Bl.	286.28	C_13_H_18_O_7_	115067	Cognitive dysfunction	–	SD	TNF-α; IL-1β; IL-6	Zheng et al. (2022b)
					Depression	0.2 g/kg	SD	CRF; TH	Zhao et al. (2023b)
					Headache	10 mmol/L	SD	CGRP; pERK1/2	Luo et al. (2011)
Chlorogenic acid	Bajiaohuixiang/*Illicium verum* Hook.f.	354.31	C_16_H_18_O_9_	1794427	Cognitive dysfunction	40 mg/kg 200 μM	C57BL/6 BV-2	Akt1; TNF; MMP9; PTGS2; MAPK1; MAPK14; RELA	Xiong (2023)
Ethanol extract	Fuling/*Poria cocos* (Schw.) Wolf	–	–	–	Dyssomnia	160 mg/kg	ICR	GABA_A_	Kim et al. (2022)
Jujubogenin	Suanzaoren/*Ziziphus jujuba* Mill. var*. spinosa* (Bunge) Hu ex H. F. Chou	472.7	C_30_H_48_O_4_	15515703	Dyssomnia	–	–	GABA_A_	Chen et al. (2008)
Piperine	Heihujiao/*Piper nigrum* L.	285.34	C_17_H_19_NO_3_	638024	Olfactory dysfunction	100 mg/kg	1-SNCA TG	SNCA; P2RX4	Li et al. (2022d)
Curcumin	Jianghuang/*Curcuma Longa* L.	368.4	C_21_H_20_O_6_	969516	Olfactory dysfunction	1 μM	OECs	TG2; PSR	Guo et al. (2020)
					Anxiety	100 mg/kg	C57BL/6	Gut-brain axis; phosphatidylcholine	Zhang et al. (2022a)
					Arrhythmia	50 μmol/L	Ventricular Myocytes of Rat	K_to_ channel; K_ir_ channel	Wang et al. (2023a)
					Arrhythmia	20 μmol/L	HEK293	hERG	Wang et al. (2023b)
					Chronic heart failure	100 mg/kg	New Zealand rabbits	DKK-3; ASK1	Cao et al. (2018)
					Irritable bowel syndrome	0.2 g/kg	SD	Gut mocrobiota	Tu et al. (2023)
Ethanol extract/E-ligustilide	Danggui/*Angelica sinensis* (Oliv.) Diels.	190.24	C_12_H_14_O_2_	5877292	Depression	7.2 g/kg	SD	PIK3CA; PIK3CD	Gong et al. (2023)
Resveratrol	Sangshen/*Morus alba* L.	228.24	C_14_H_12_O_3_	445154	Fatigue	10 mg/kg	Mice	TP53; PIK3R1; AKT1; PIK3CA; MAPK1	Ma et al. (2023)
					Arrhythmia	100 μM	Rat cardiomyocytes	Calcium channel	Zhang et al. (2023e)
Ethanol extract	Roucongrong/*Cistanche deserticola* Y. C. Ma	–	–	–	Cancer-related fatigue	500 mg/kg 250 μg/mL	BALB/c C2C12	Beclin-1; HIF-1α; BNIP3	Zhang et al. (2023d)
Quercetin	Bajiaohuixiang/*Illicium verum* Hook.f.	302.23	C_15_H_10_O_7_	5280343	Pulmonary fibrosis	100 mg/kg	C57BL/6	SphK1; S1PL	Zhang et al. (2018)
					Pulmonary fibrosis	–	–	AKT1, PIK3CG	Zhang et al. (2021)
Hesperidin	Juhua/*Chrysanthemum morifolium* Ramat.	610.6	C_28_H_34_O_15_	10621	Pulmonary fibrosis	200 mg/kg 50 μg/mL	C57BL/6 MRC-5	Cell cycle regulatory proteins; α-SMA	Han et al. (2023)
					Pulmonary fibrosis	100 mg/kg	SD	Nrf2; HO-1; TNF-α; IL-1β; IL-6; TGF-β; Smad-3; NF-κB; IκBα; AMPK; PP2C-α	Zhou et al. (2019)
Platycodin D	Jiegeng/*Platycodon grandiflorum* (Jacq.) A. DC.	1225.3	C_57_H_92_O_28_	162859	Pulmonary fibrosis	10 mg/kg 10 μmol/L	C57BL/6 Primary Pulmonary Fibroblasts	α-SMA; collagen Ⅰ; TRPC6	Liang et al. (2024)
Sodium Houttuyfonate	Yuxingcao/*Houttuynia cordata* Thunb.	302.36	C_12_H_23_NaO_5_S	23663544	Pulmonary fibrosis	90 mg/kg	KM	IL-1β; IL-6; TNF-α; TGF-β1	Shen et al. (2021)
Total flavonoids	Huangqi/*Astragalus membranaceus* (Fisch.) Bge. var. *mongholicus* (Bge.) Hsiao	–	–	–	Pulmonary fibrosis	50 mg/kg 100 μg/mL	C57BL/6 A549	TGF-β1; Wnt7b	Yang et al. (2023a)
Astragaloside IV	Huangqi/*Astragalus membranaceus* (Fisch.) Bge. var. *mongholicus* (Bge.) Hsiao	785	C_41_H_68_O_14_	13943297	Pulmonary fibrosis	20 mg/kg 100 μg/mL	Rats A549	TGF-β1; FOXO3a	Qian et al. (2018)
					Pulmonary fibrosis	20 mg/kg	SD	Collagen I, fibronectin and α-SMA	Li et al. (2021b)
					Pulmonary fibrosis	20 mg/kg 10 μM	C57BL/6 BEAS-2B	RAS	Zhang et al. (2023a)
					Pulmonary fibrosis	20 mg/kg 100 μg/mL	SD A549	lncRNA-ATB; miR-200c; ZEB1	Guan et al. (2024b)
					Cardiac protection	80 mg/kg	SD	TLR4; MyD88; NF-κb; TGF-β	Shi et al. (2021)
					Cardiac protection	40 mg/kg	C57BL/6	Nrf-2; HO-1	Chen et al. (2023)
					Cardiac protection	12.5 μM	H9c2	HIF-1α	Li et al. (2021a)
					Cardiac protection	–	–	sarcoplasmic reticulum Ca^2+^ pump	Zang et al. (2020)
Puerarin	Gegen/*Pueraria lobata* (Willd.) Ohwi	416.4	C_21_H_20_O_9_	5281807	Arrhythmia	1.2–9.6 mmol/L	Rat Ventricular Myocyte	K_1_ channel	Wang et al. (2023a)
					Hypertension	80 mg/kg	SHR, WKY	eNOS; AT1; Cav1	Shi et al. (2019)
					Irritable bowel syndrome	24 mg/kg	SD	HPA axis; CRF1	Wang et al. (2021)
Formononetin	Huangqi/*Astragalus membranaceus* (Fisch.) Bge. var. *mongholicus* (Bge.) Hsiao	268.26	C_16_H_12_O_4_	5280378	Cardiac protection	40 mg/kg 10 μM	C57BL/6 Cardiomyocyte	ALDH2; HADH; MOAB	Qian et al. (2024)
Calycosin	Huangqi/*Astragalus membranaceus* (Fisch.) Bge. var. *mongholicus* (Bge.) Hsiao	284.26	C_16_H_12_O_5_	5280448	Cardiac protection	50 mg/kg 40 μM	C57BL/6 Primary Cardiac Fibroblasts	TGFBR1; Smad2/3	Chen et al. (2022a)
Polysaccharide	Gouqizi/*Lycium barbarum* L.	–	–	–	Vascular disease	4 mg/mL	Vascular smooth muscle primary cell	PI3K; Akt; miR-145	Zhang et al. (2020b)
					Reproduction regulation	1.5 g/kg	KM	IL-2; IL-12; TNF-α	Qian (2019)
					Reproduction regulation	20 mg/kg	Immp2l mutant mice	GPX4; AIF	Liu et al. (2021)
					Reproduction regulation	40 mg/kg	Immp2l mutant mice	TNP2; RPL31	Liu et al. (2020a)
					Reproduction regulation	50 mg/kg	SD	Fas	Zhou et al. (2022a)
Anthocyanin isomers	Gouqizi/*Lycium barbarum* L.	–	–	–	Hypertension	–	–	ACE	Lu et al. (2023)
Uncarialin A	Pugongying/*Taraxacum mongolicum* Hand. -Mazz.	–	–	–	Hypertension	10 mg/kg	C57BL/6	Cav1.2	Yun et al. (2020)
6-gingerol	Shengjiang/*Zingiber officinale* Rosc.	294.4	C_17_H_26_O_4_	442793	Nausea; Vomiting	–	Wistar guinea pig	5-HT3; NK1; M3	Pertz et al. (2011), Haniadka et al. (2012)
8-gingerol	Shengjiang/*Zingiber officinale* Rosc.	322.4	C_19_H_30_O_4_	168114	Nausea; Vomiting	–	Wistar guinea pig	5-HT3; NK1; M3	Pertz et al. (2011), Haniadka et al. (2012)
10-gingerol	Shengjiang/*Zingiber officinale* Rosc.	350.5	C_21_H_34_O_4_	168115	Nausea; Vomiting	–	Wistar guinea pig	5-HT3; NK1; M3	Pertz et al. (2011), Haniadka et al. (2012)
6-shogaol	Shengjiang/*Zingiber officinale* Rosc.	276.4	C_17_H_24_O_3_	5281794	Nausea; Vomiting	–	Wistar guinea pig	5-HT3; NK1; M3	Pertz et al. (2011), Haniadka et al. (2012)
Polysaccharide	Fuling/*Poria cocos* (Schw.) Wolf	–	–	–	Irritable bowel syndrome	–	Fresh feces	Gut microbiota	Yang et al. (2023b)
Water extract	Rougui/*Cinnamomum cassia* Presl	–	–	–	Irritable bowel syndrome	270 mg/kg 4,000 μg/mL	SD HEK293	Tph1; 5-HT	Yu et al. (2022)
Aqueous extract	Yuxingcao/*Houttuynia cordata* Thunb.	–	–	–	Gut regulation	400 mg/kg	C57BL/6	ZO-1; IL-10; IL-1β; IL-6; EGFR	Wang et al. (2022a)
Flavonoid	Gancao/*Glycyrrhiza uralensis* Fisch.	–	–	–	Gastric ulcer	300 mg/kg 2.5–15 μg/mL	SD GES-1	MUC5AC; MUC6; gut microbiota	Wu et al. (2023)
Ginsenoside Rg1	Xiyangshen/*Panax quinquefolium* L.	801	C_42_H_72_O_14_	441923	Reproduction regulation	40 mg/kg 50 μM	C57BL/6 GC-2spd (ts)	Akt; GSK-3β; NRF2	Wang et al. (2023b)
Volatile oil	Shengjiang/*Zingiber officinale* Rosc.	–	–	–	Alopecia	–	–	TNF; IL6; ALB	Pan et al. (2022)
Water extract	Xiebai/*Allium macrostemon* Bge.	–	–	–	Alopecia	3% (w/v) 30 μg/mL	C57BL/6 HDPC	β-catenin, p-GSK-3β, Cyclin D1	Gao et al. (2023)
Polysaccharide	Tiepishihu/*Dendrobium officinale* Kimura et Migo	–	–	–	Alopecia	5 g/L (200 μL)	C57BL/6	β-catenin; cyclin D1; 5α-reductase	Zhang et al. (2023f)
Extract (10% flavonoid)	Pugongying/*Taraxacum mongolicum* Hand. -Mazz.	–	–	–	Rash	0.5 g mix with 200 μg deionized water 5 μg/mL	New Zealand Rabbit Human Dermal Fibroblasts	IL-6; IL-8	Zhang et al. (2023b)

^a^ Data are derived from Pubchem database (https://pubchem.ncbi.nlm.nih.gov/). OECs, olfactory ensheathing cells; HDPC, human hair dermal papilla cell; TNF-α, tumor necrosis factor alpha; IL-1β, interleukin 1β; IL-6, interleukin 6; CRF, corticotropin-releasing factor; TH, tyrosine hydroxylase; pERK1/2, phosphorylated extracellular signal-regulated kinase1/2; CGRP, calcitonin gene-related peptide; Akt1, serine/threonine kinase 1; TNF, tumor necrosis factor; MMP9, matrix metallopeptidase 9; PTGS2, prostaglandin-endoperoxide synthase 2; MAPK1, mitogen activated protein kinase1; MAPK14, mitogen-activated protein kinase 14; GABA_A_, gamma aminobutyric acid type A; SNCA, Α-synuclein; P2RX4, purinergic receptor P2X ligand-gated ion channel 4; TG2, transglutaminase-2; PSR, phosphatidylserine receptor; hERG, human-ether-a-go-go-related channel; DKK-3, Dickkopf-related protein 3; ASK1, apoptosis signal-regulating kinase 1; PIK3CA, phosphatidylinositol-4,5-bisphosphate 3-kinase catalytic subunit alpha; PIK3CD, phosphoinositide 3-kinase catalytic subunit delta; TP53, tumor suppressor gene p53; PIK3R1, phosphoinositide-3-kinase regulatory subunit 1; HIF-1α, hypoxia inducible factor1α; BNIP3, Bcl-2/E1B-19k-interacting protein 3; SphK1, sphingosine kinase 1; S1PL, sphinogosine-1-phosphate lyase; PIK3CG, phosphatidylinositol-4,5-bisphosphate 3-kinase catalytic subunit gamma; α-SMA, α-smooth muscle actin; Nrf2, nuclear factor E2-related factor 2; HO-1, heme oxygenase-1; TGF-β, transforming growth factor-beta; NF-κB, nuclear factor-Kappa B; IκBα, inhibitorα of NF-κB; AMPK, adenosine monophosphate-activated protein kinase; PP2C-α, serine/threonine protein phosphatase type 2C alpha; TRPC6, transient receptor potential cation channel subfamily C member 6; FOXO3a, forkhead box O3a; ZEB1, zinc finger E-box-binding homeobox 1; TLR4, toll-like receptor 4; MyD88, myeloid differentiation factor 88; eNOS, endothelial nitric oxide synthase; AT1, angiotensin II type 1-receptor; Cav1, caveolin-1; HPA, hypothalamic-pituitary-adrenal; CRF1, corticotropin-releasing hormone receptor 1; ALDH2, aldehyde dehydrogenase 2; HADH, mitochondrial enzyme β-hydroxyacyl-CoA-dehydrogenase; MOAB, monoamine oxidase B; TGFBR1, transforming growth factor-beta receptor 1; PI3K, phosphoinositide 3-kinase; IL-2, interleukin 2; IL-12, interleukin 12; GPX4; glutathione peroxidase 4; AIF, apoptosis-inducing factor; TNP2, transition protein 2; RPL31, ribosomal protein L31; ACE, angiotensin converting enzyme; Cav1.2, L-type calcium channel subunit alpha-1C; 5-HT3, 5-hydroxytryptamine; NK1, neurokinin-1; M3, muscarinic acetylcholine receptor M3; Tph1, tryptophan hydroxylase 1; 5-HT, serotonin; ZO-1, zonula occludens-1; IL-10, interleukin-10; EGFR, epidermal growth factor receptor; MUC5AC, mucoprotein 5AC; MUC6, mucoprotein 6; GSK-3β, glycogen synthase kinase 3β; NRF2, NF-E2-related factor 2; Akt, protein kinase B; ALB, albumin; IL-8, interleukin-8.

## 5 Regulatory characteristic of FMH food

Huangqi is one of the common FMH foods, containing multiple active ingredients (such as astroloside IV, formononetin, and calycosin) that interfere with many PACS symptoms (Xu Lou et al., 2024). This intervention mechanism involves multiple metabolites, multiple targets, and multiple pathways, which is the most prominent feature of FMH foods. Here, this complex regulatory mechanism is exemplified by the role of Huangqi in cardiac protection. Astragaloside IV, a main ingredient of Huangqi, is a type of cycloartane-type triterpene glycoside, which can interfere with cardiac hypertrophy, ischemia reperfusion injury, hypertension, atherosclerosis, chronic heart failure, and other cardiovascular diseases (Tan et al., 2020; Zhang J. et al., 2020; Li M. et al., 2022; Xu Lou et al., 2024). Formononetin is a methoxy isoflavone with many activities, such as antioxidant, cardioprotective, vasodilator, and anti-inflammatory (Wang D. S. et al., 2020; Machado Dutra et al., 2021). Calycosin is an isoflavone with antioxidant, anti-apoptotic, anti-inflammatory properties, promoting angiogenesis (Deng et al., 2021; Chen G. et al., 2022). In terms of cardiac protection, these three metabolites mainly regulate four signaling pathways: TLR4/MyD88/NF-κB, TGF-β/Smad, Nrf-2/HO-1, and PI3K/AKt (Li M. et al., 2022; Xu Lou et al., 2024).

TLR4 activates NF-κB through MyD88 to participate in the inflammatory response and involves the release of chemotactic factors and the expression of endothelial cell adhesion factors (Li and Wang, 2017). Studies have shown that the regulation of TLR4/MyD88/NF-κB is involved in the induction of acute cardiovascular disease-related processes, such as infiltration and activation of immune cells, formation of lipid cores, reduction of fibrous cap thickness, and plaque rupture (Li and Wang, 2017; Zhang Q. et al., 2022). Astragaloside IV could significantly reduce the expression of TLR4, MyD88, and NF-κB p65, and inhibit the occurrence and development of related inflammation (Shi et al., 2021). Studies have shown that formononetin and calycosin mediate the death and inflammation of microglia induced by oxygen-glucose deprivation/reperfusion by regulating TLR4/MyD88/NF-κB pathway, and reduce cerebral ischemia-reperfusion injury (Li Y. et al., 2023; Chen et al., 2024). These studies provide important references for myocardial cells exhibiting similar or same symptoms (Krech et al., 2017).

In addition, TLR4 can also enhance the effect of TGF-β (Mzyk et al., 2022). TGF-β/Smad pathway is involved in the regulation of fibrosis-related gene expression, such as proteoglycan, collagen, matrix metalloproteinase (Shang and Zhou, 2020). In addition to intervening fibrosis, TGF-β can also influence macrophage-associated anti-inflammatory transition, fibroblast-associated reparative scar formation, and pericyte-associated maturation of infarcted neovessel (Frangogiannis, 2024). Studies have shown that electroacupuncture stimulation combined with astragaloside IV treatment can effectively alleviate isoproterenol-induced cardiac hypertrophy and myocardial fibrosis in rats, and this regulation is involved in the TGF-β/Smad pathway (Li J. S. et al., 2017). Chen G. et al. (2022) showed that calycosin could alleviate myocardial fibrosis and cardiac dysfunction after myocardial infarction *in vivo* by inhibiting TGF-β/Smad pathway, and inhibit cardiac fibroblasts proliferation and collagen deposition induced by TGF-β1 *in vitro*.

HO-1 plays an important role in maintaining cardiac homeostasis and cardiac injury repair, and the defense mechanism of HO-1 driven by Nrf2 has the protective effect of myocardium (Wang J. et al., 2020). The Nrf2/HO-1 pathway can also regulate calcium levels and interfere with the occurrence of pyroptosis, autophagy, and programmed cell necrosis (Zhang X. et al., 2020). Furthermore, HO-1 can also inhibit the production, myocardial infiltration, and inflammatory properties of monocytes and macrophages, and interfere with cardiac injury caused by myocardial infarction (Tomczyk et al., 2019). Chen et al. (2023) have reported that astragaloside IV could inhibit NLRP3 inflammasome-mediated pyroptosis by activating the Nrf-2/HO-1 pathway and intervene the doxorubicin-induced cardiac dysfunction. Both formononetin and calycosin have also been shown to be involved in the regulation of Nrf2/HO-1 pathway and interfere with oxidative stress and inflammation (Aladaileh et al., 2019; Liu et al., 2022). However, the roles that formononetin and calycosin play in cardiac protection have not been reported.

**FIGURE 3:**
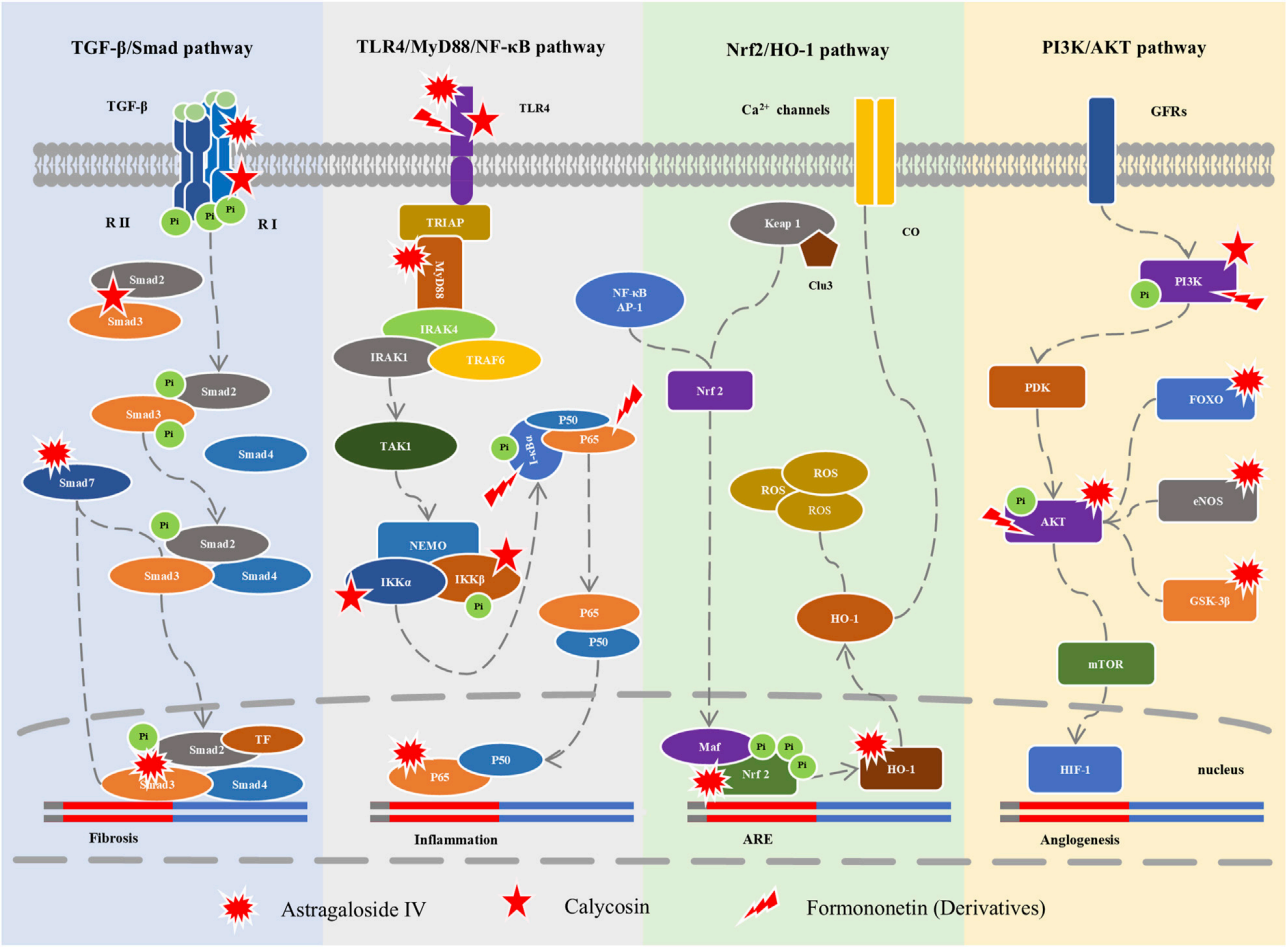
Regulation of pathways related to cardiac protection by metabolites of Huangqi.

PI3K/AKT pathway is involved in the regulation of cell cycle, growth, and proliferation (Ghafouri-Fard et al., 2022). It is also involved in cardiac remodeling, repair, angiogenesis, and inflammatory response (Ghafouri-Fard et al., 2022; Zhang Q. et al., 2022), and interfering with the occurrence and development of cardiac fibrosis and regulating the expression of downstream related genes, e.g., phosphorylated PI3K and Akt inhibit the expression of mTOR and FOXO (Qin et al., 2021; Zhang Q. et al., 2022). Studies have shown that astragaloside IV regulated the expression of Nrf2, Bach1, and HO-1, inhibited hypoxia-reoxygenation-induced H9c2 cardiomyocyte injury (Yang et al., 2019), reduced the myocardial ischemia-reperfusion injury by regulating the PI3K/AKT/GSK-3β pathway (Wei et al., 2019), and induced the dilation of the aortic rings by up-regulating the expression of eNOS mRNA (via PI3K/Akt/eNOS pathway) (Lin et al., 2018). Luo and Pan (2024) reported a formononetin derivative, which interfered with apoptosis and autophagy and reduced myocardial enzyme activity and the number of autophagic vesicles. Calycosin has been used as a PI3K activator to reduce inflammation and fibrosis symptoms caused by heart failure through the AKT-IKK/STAT3 axis (Wang J. et al., 2022). The complex mechanisms involved in this section are demonstrated in Figure 3.

## 6 Safety of FMH food

Safety is an important topic in the application and development of FMH foods. Compared with conventional drugs, these foods have a higher level of safety. However, the FMH idea does not imply that there is no distinction between medicine and food, while people should remain aware of their differences during use (Xie et al., 2020). It is noted that clear and explicit requirements are enforced on the inclusion of the total 105 FMH foods acknowledged by the National Health and Health Commission of the People’s Republic of China. These FMH foods, traditionally used as food and included in the *Chinese Pharmacopoeia*, have passed safety assessment and conform to relevant regulations (Chen, 2023; Li S. et al., 2024). However, there are still some issues that require attention during their use. These issues are discussed in this section.

### 6.1 Differences in the scope of FMH food collection between ancient and modern times

The scope of the definition of FMH food in ancient and modern China is different (Fan, 2016). For example, Renshen (*Panax ginseng* C. A. Mey.), considered a type of FMH food in the Tang Dynasty and a type of medicine in the Song Dynasty, is not included in the FMH food list acknowledged by the National Health and Health Commission of the People’s Republic of China. Similarly, many botanical drugs used in some FMH food prescriptions were considered FMH foods in ancient times but are no longer classified as food in modern times. By considering the items listed as food in “*Beiji Qianjin Yaofang Shizhi*” and “*Shiliao Bencao,*” and those listed as medicine in “*Xinxiu Bencao,*”, it was found that Cangerzi (*Xanthium strumarium* L.) appeared in all three texts. Therefore, it was regarded as an FMH food during the Tang Dynasty (Fan, 2016). However, Cangerzi is not included in the modern list of FMH foods.

### 6.2 Toxicity of improper consumption

According to the definition of FMH food, the FMH foods are also Chinese medicinal materials, with characteristics of complex metabolites (including both active ingredients and toxic metabolites). Studies have shown that improper consumption of these foods could cause damage to the kidney, liver, and heart, and even death, e.g., gardenoside in Zhizi (*Gardenia jasminoides* J. Ellis) and extract of Juemingzi (*Cassia obtusifolia* L.) (Shi and Jiang, 2020). It is reported that the safe dose of Zhizi is 6–10 g, whereas the consumption of 30 g induces significant liver toxicity (Yang et al., 2022) and a single intragastric administration of a specific dose of Juemingzi extract in mice has been shown to cause severe liver and kidney toxicity (Huang et al., 2016; Huang et al., 2017).

### 6.3 Principles of food incompatibility

In addition to the toxicity of the single FMH food, there are also restricted taboos in the combination of multiple foods. At present, the well recognized theories of incompatibility in TCM are called “*Eighteen Fan*” (i.e., eighteen incompatible medicaments, including Gancao-Gansui, Daji, Haizao, and Yuanhua; Wutou-Beimu, Gualou, Banxia, Bailian, and Baiji; Lilu-Renshen, Shashen, Danshen, Xuanshen, Xixin, and Baishao) and “*Nineteen Wei*” (i.e., nineteen medicaments of mutual restraint, including Liuhuang-Poxiao, Shuiyin-Pishuang, Langdu-Mituoseng, Badou-Qianniu, Dingxiang-Yujin, Chuanwu-Xijiao, Caowu-Xijiao, Yaxiao-Sanleng, Guangui-Shizhi, and Renshen-Wulingzhi) (Li et al., 2019). The “*Fan*” and “*Wei*” refer to the facts that the combined application of these drugs will either weaken the efficacy or enhance the side effects; the occurrence of this situation are probably attributed to the addition of toxic metabolites, the change of pharmacological and toxicological reactions, and the change of drug metabolism (Wei, 2013; Ou et al., 2016; Tian et al., 2023). However, these theories are not absolute. Under certain conditions, some drugs can be used simultaneously, and combination of drugs not mentioned as incompatible in the theory may not be absolutely safe, and further confirmation is needed (Wei, 2013; Yan, 2013).

### 6.4 Food toxicology and xenobiotics

In our daily life, the toxicity of FMH food is also influenced by xenobiotics, such as derivatives, e.g., heterocyclic aromatic amines (HAAs), benzo(a) pyrene (BaP), and advanced glycation end products (AGEs) (Barzegar et al., 2019; Fu et al., 2022; Shi et al., 2024), pollutants (e.g., pesticides, heavy metals, and antibiotics) (Ding et al., 2021), and additives (e.g., preservatives, sweeteners, and pigments) (Rinninella et al., 2020).

### 6.5 Quality control and experimental evaluation

At present, there are still significant challenges of quality control in the research field of FMH foods. Different origins, cultivation conditions, and processing technologies could cause the variations in the quality of FMH foods (Yao et al., 2024; Xiong et al., 2025), which could be improved by the application of molecular fingerprint and characteristic spectrum analyses (Xiong et al., 2025). Furthermore, some efficacy metabolites of FMH foods demonstrated in current studies have not been tested *in vivo* or have only been proved to be effective in theory. Moreover, these studies have mainly focused on pharmacological and toxicological effects, with limited information available regarding their quality and safety risks (Qiu et al., 2025). Therefore, we should maintain a cautious attitude when using FMH foods, consuming them under the guidance of professionals, relevant laws and regulations, and the approved list of FMH substances, to prevent the occurrence of medicine/food-induced diseases.

## 7 Conclusion

The end of COVID-19 pandemic has not brought ideal peace to the world, and PACS in the post-epidemic era is still threatening human health. The long-term and multi-system characteristics of PACS indicate the arrival of the era of long-term management of COVID-19. The thought of FMH is a kind of food therapy derived from TCM. Although FMH foods are homologous to TCM botanical drugs and other animal-based traditional Chinese medicines, they have the advantage of distinguishing between medicine and regular food. They are considered relatively safe chronic disease intervention drugs and demonstrated to play an important role in the long-term management of PACS through daily consumption. In this review, the intervention effects of metabolites in FMH foods on PACS are summarized, and their medicinal values and application potentials are fully demonstrated and clarified. However, due to the relatively new nature of this field and the lack of targeted results, some application examples shown in this paper may partially broaden the scope of PACS symptoms (i.e., the difference between symptoms was not strictly defined), and they also serve extensive summaries of intervention examples of PACS-related symptoms or similar symptoms (including those not caused by COVID-19). These symptoms share similarities with PACS in terms of pathogenesis and intervention mechanism, therefore, such examples—IPF and PF caused by PACS—can also serve as references for relevant studies.

Numerous studies have shown that FMH foods and their active metabolites play important roles in PACS-related multi-systems and multi-symptoms. However, the actual contents and nutritional mechanisms of many FMH food metabolites summarized in this review have not been fully investigated, and sufficient evidence is still lacking to support the findings revealed in these studies. For example, the extensive and magical efficacy of quercetin and kaempferol, which are “star molecules” in TCM, still needs to be further explored. The dietary characteristics of FMH foods provide them with significant advantages over drugs, whereas these do not obscure or alter their fundamental nature as Chinese medicinal materials. People should pay special attention to dosage limits, potential toxicity, and combination when consuming these foods in daily life. Furthermore, as valuable complementary medical materials, FMH foods and their metabolites could help alleviate symptoms to some extent, but may not achieve the desired therapeutic effect, and patients still require medical assistance and professional guidance when necessary.

This field is relatively new and still facing such common challenges as the lack of clinical evidence and targeted research as well as the determination of toxic doses. Currently, most research in this field focuses on pharmacological analysis, and further investigations should focus on the clinical applications of the FMH foods. The use of computation technologies, such as network pharmacology and molecular docking, have facilitated and advanced the analysis of FMH food mechanism to provide more evidence to verify the findings revealed in the relevant studies. For example, it remains unclear whether each metabolite plays a role through the targets and pathways identified by the network analysis. In addition, it is noted that the efficacy and mechanisms of these metabolites in FMH foods are obtained through cell and animal experiments. Although the theoretical limitations have been addressed, clinical experiments are necessary to validate the findings based on cell and animal studies.

## 8 Prospects

With the increasing demand for healthy functional foods and the continuous research on the medicinal and nutritional mechanisms of FMH foods, the development of new foods that take into account high nutrition and safety has increasingly become an important topic in the field of food science. Effective and economical experimental methods are an important factor to promote the high-quality development of the FMH food industry. In recent years, with the vigorous development of bioinformatics, network pharmacology, virtual screening, high-throughput sequencing, machine learning, and dynamic simulation, these technologies have gradually integrated into the field of food science. They have played important roles in the investigations of food metabolite collection, potential target screening, drug mechanism elucidation, compatibility rule analysis, and new drug discovery, and reduced the cost of food development.

Therefore, we anticipate that future research on PACS intervention through food can focus on the following five aspects:

(1) To establish a comprehensive metabolite and target database of FMH foods, utilizing bioinformatics and advanced methods to explore nutritional mechanisms at both the system and molecular levels, with a focus on system nutrition and precision nutrition.
(2) To conduct safety evaluation and taste assessments of the developed food therapy programs or food prescriptions, based on the compatibility principles of TCM, food toxicology indices, and sensory evaluation requirements.
(3) To conduct data mining on existing ingredient tables of medicinal diets or healthcare foods, explore their combination rules, and identify alternative materials with higher economic benefits, broader applicability, greater nutritional values, and improved sensory qualities.
(4) To verify the beneficial effect of daily intake of Jianghuang as a spice on PACS patients. This selection is based on a review of relevant studies, suggesting that curcumin in Jianghuang could be a potential metabolite for intervention of PACS, due to its action on multiple systems and its role in treating COVID-19-related OD, anxiety, PF, arrhythmia, and myocardial injury (Guo et al., 2020; Wang L. et al., 2022; Zhang F. et al., 2022; Wang C. et al., 2023; Yang C. et al., 2024).
(5) To focus on the analysis of the active components in the Chinese medicine extracts. With the technical support of theoretical methods, such as network pharmacology, in drug screening, the mechanisms underlying the therapeutic and application effects of the drugs should be comprehensively explored through cell, animal, and clinical experiments, rather than being limited to simple pharmacological analysis.

In conclusion, we believe that our review provides important references for the development of FMH foods, which could serve potential drugs for intervention of PACS in the post-epidemic era.

## Glossary

**FMH**: Food and medicine homology
**SARS-CoV-2**: coronavirus 2
**COVID-19**: corona virus disease 2019
**PACS**: post-acute COVID-19 syndrome
**FMH**: food and medicine homology
**WHO**: World Health Organization
**PHEIC**: Public Health Emergency of International Concern
**ACE2**: angiotensin-converting enzyme 2
**TCM**: traditional Chinese medicine
**TMPRSS2**: transmembrane serine protease 2
**EBV**: Epstein Barr virus
**HHV-6**: human heroes type 6
**BBB**: blood brain barrier
**CNS**: central nervous system
**MCAS**: mast cell activation syndrome
**Ang II**: angiotensin II
**ROS**: reactive oxygen species
**OD**: olfactory dysfunction
**PNS**: peripheral nervous system
**OE**: olfactory epithelium
**α-syn**: α-synuclein
**PD**: Parkinson’s disease
**OB**: olfactory bulb
**DAD**: diffuse alveolar damage
**ECM**: extracellular matrix
**PF**: pulmonary fibrosis
**ICU**: intensive care unit
**IPF**: idiopathic pulmonary fibrosis
**EMT**: epithelial-mesenchymal transition
**POTS**: postural orthostatic tachycardia syndrome
**CCK**: cholecystokinin
**GLP-1**: glucagon-like peptide-1
**SCFAs**: short-chain fatty acids
**LPS**: lipopolysaccharide
**IBS**: irritable bowel syndrome
**AKI**: acute kidney injury
**CKD**: chronic kidney disease
**PTC**: proximal tubular cells
**ED**: erectile dysfunction
**ARDS**: acute respiratory distress syndrome
**NDDs**: neurodegenerative diseases
Other target abbreviations are listed in the notes of Table 3.
